# Alignment-free genomic sequence comparison using FCGR and signal processing

**DOI:** 10.1186/s12859-019-3330-3

**Published:** 2019-12-30

**Authors:** Daniel Lichtblau

**Affiliations:** Wolfram Research, Champaign, 61820 Illinois USA

**Keywords:** Alignment-free methods, Genome comparison, Genome identification, Chaos game representation, Phylogenetic tree, Dimension reduction

## Abstract

**Background:**

Alignment-free methods of genomic comparison offer the possibility of scaling to large data sets of nucleotide sequences comprised of several thousand or more base pairs. Such methods can be used for purposes of deducing “nearby” species in a reference data set, or for constructing phylogenetic trees.

**Results:**

We describe one such method that gives quite strong results. We use the Frequency Chaos Game Representation (FCGR) to create images from such sequences, We then reduce dimension, first using a Fourier trig transform, followed by a Singular Values Decomposition (SVD). This gives vectors of modest length. These in turn are used for fast sequence lookup, construction of phylogenetic trees, and classification of virus genomic data. We illustrate the accuracy and scalability of this approach on several benchmark test sets.

**Conclusions:**

The tandem of FCGR and dimension reductions using Fourier-type transforms and SVD provides a powerful approach for alignment-free genomic comparison. Results compare favorably and often surpass best results reported in prior literature. Good scalability is also observed.

## Background

For fairly short nucleotide sequences, of up to perhaps 100 bp, methods that are based on aligning strings can be quite powerful. These do not scale well to longer sequences. In recent decades there has thus been considerable work in developing alignment-free methods for comparing longer gene fragments. A far from exhaustive list of references is [1–31] (and an extensive review of these is found in [32]). A key idea is to capture some aspects of the sequences, perhaps as images or numeric vectors, and apply image and/or signal processing methods in a way that is fast and allows for distance-based comparisons. One family of methods (well represented in the above references) uses the Frequency Chaos Game Representation (FCGR) [2, 7] (based on earlier work by Jeffrey [14]). This creates images with certain fractal properties that capture frequencies of *k*-mers for modest values of *k* (as will be explained in the “Frequency chaos game representation” subsubsection of the “Implementation” section). A number of different processing methods have then been deployed in order to classify these images; references [2, 7, 12, 15, 16, 25, 28–30] show several of these and convey some idea of their variety.

The approach we will take starts with these FCGR images. We use a Fourier Discrete Cosine Transform (DCT) of each image matrix, retaining only low frequency components in order to reduce dimension. We then flatten the resulting matrices into vectors and use the Singular Value Decomposition (SVD) to further reduce dimension. The vectors that result from this can be used in several ways. We will put them into a kd tree [33] for purposes of finding nearby sequences. This can be applied to inferring species or other taxonomy information for new sequences, given a reference database for known genomes [27, 28, 30]. We also use them to train neural networks. Another use we will show is in hierarchical clustering e.g. to create a phylogenetic tree [12, 21, 24, 27, 30]. A related application we will cover is to infer bacterial hosts from viral genomes [1, 8].

This method of working with images is by no means new. Fourier and SVD based methods have been in use for at least two decades, and have been used in tandem as well [34]. A particularly effective approach, also using kd trees for lookup, is described in [35]. This previous work gave an example involving FCGR images, which serves as a proof-of-concept from which the present work arose. Here we describe several refinements that are specific to working with FCGR images from nucleotide sequences, that improve accuracy while maintaining computational efficiency. We also describe several variations, indicating strengths and weaknesses thereof. One strength we should note at the outset is that there is no requirement that nucleotide sequences have a common length (as is sometimes the case for alignment-free methods that use signal processing). Indeed, some experiments will involve sequences of quite different lengths.

Other related methods involve use of a Fourier transform, or similar, on signal vectors created from a CGR, from indicator vectors, or by any of several related means. A number of these methods were developed by Yau and coauthors (see [12, 21, 25, 26] and references therein). These were used with considerable success to deduce phylogeny trees from genome sequences; some of their tests are now benchmarks. A powerful set of related methods also appears in [[27], with strong result shown for several tests both in species recognition and phylogeny tree construction (which the authors have made available for benchmark purposes). The tandem of FCGR and SVD is used in [29] on a set of 400 of Human Papillomavirus (HPV) genomes from 12 strains, where it attains perfect classification at the strain level (this data set was also handled quite well in [12, 26]).

All tests herein were run with version 12 of Mathematica [36]. Tests were run on a desktop machine with a 3 GHz processor, 16 Gb RAM, running under the Linux operating system. The full Wolfram Language code for all experiments is available in the Additional file 1.

## Implementation

### Transforming genome sequences to short vectors

We start with descriptions of the steps to our approach. The main steps are as follows. The Frequency Chaos Game Representation converts nucleotide strings to images. The Discrete Cosine Transform reduces the dimension of these images. The Singular Values Decomposition further reduces dimension so that we can put vectors it produces into a searchable data structure. We mention some variants as we proceed. The hyperparameters used in each step have been selected based on results from running many experiments with the data sets that will be described later. These seem to be stable values insofar as small changes do not give large changes in outcomes. We note that there are usually trade-offs involved in stronger dimension reduction vs. accuracy of results.

#### Frequency chaos game representation

The idea behind the Chaos Game Representation is quite simple. It starts by labeling a 1-by-1 square with a distinct nucleotide in each corner (it is common to put place purines diagonally across from one another and likewise pyrimidines, but other placements have been used). Beginning in the center, one places a dot halfway from there to the corner corresponding to the first nucleotide in the sequence. Continuing from that point, one places a second dot halfway toward the corner corresponding to the next nucleotide, and continues in this manner until the string is exhausted.

Given a positive integer *k*, if we form pixels at granularity of 2^*k*^ then Jeffrey showed that each dot corresponds to a specific character string of length *k* [14]. This in turn gave rise to a faster computational stratagem, the FCGR, as employed in [2, 7, 16]. It can be shown that each pixel corresponds to a particular *k*-mer. That is to say, a pixelation level of 7, for example, means that each pixel corresponds uniquely to a length 7 oligonucleotide, and thus occurrences of all oligonucleotides can be enumerated. The FCGR is a visual way of tallying occurrences of each *k*-mer by relative lightening of corresponding pixels. We do a nonlinear rescaling in order to get an average value that is not too close to white or black. A rescaling that has worked well in practice is to take the (real) fifth root of each pixel value, after first normalizing so that the maximum value is unity.

**Fig. 1:**
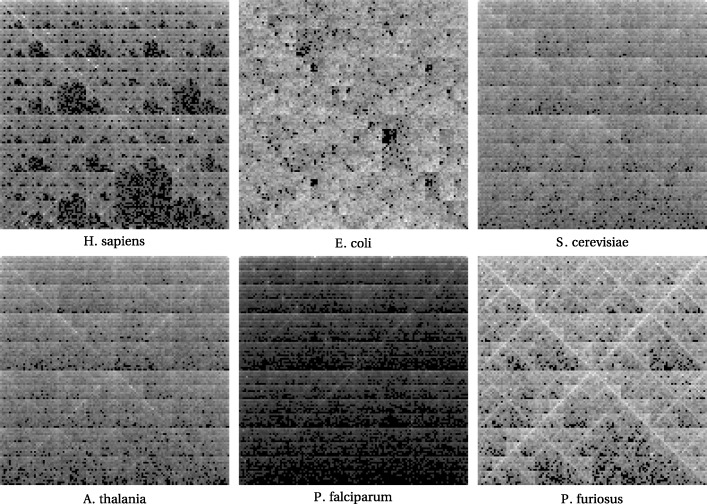
FCGR images for six distinct species

We show images in Fig. 1 created from initial nucleotide sequences of length 150000 bp from the following species: H. sapiens, E. coli, S. cerevisiae, A. thalania, P. falciparum, and P. furiosus. These were done at pixelation of 7, so the images are 128x128. These were made by the author for [35].

For the purposes at hand we have found that a pixelation level in the range of 7 to 10 tends to work well for purposes of locating nearby species in a reference set to a given gene fragment. All experiments used level 7 as that seemed to be most suitable overall; it gives good results while not needing so much memory as the higher levels.

#### Discrete cosine transform

We begin with a motivation for having two levels of dimension reduction. The second one, using a singular values decomposition, operates at the matrix level. The row dimension is the number of data points, while the column dimension is the length of each data point. The time complexity and memory requirements of this operation (which we describe in more detail later) both correlate with this column count. If we do no prior reduction, the vectors in the matrix will be comprised of flattened FCGR image values. As we work with a pixelation level of 7, these matrices are 128×128, and the column dimension would therefore be 128^2^, or 16384. For large data sets this would impose a considerable memory requirement on the SVD step, as well as have a large speed cost. We can avoid this by reducing image sizes one at a time, so there is no large matrix constructed, hence no large memory requirement.

The Discrete Fourier Cosine Transform (DCT) provides a useful tool for exactly this purpose. It is a real-valued variant of the Discrete Fourier Transform that is known to have good properties for concentrating most of the spectral energy of images into the low frequency components. It thus provides good image fidelity while allowing to reduce memory usage considerably. This is key to obtaining a matrix of manageable column dimension, for purposes of further dimension reduction. Empirically it has been found that, for this purpose, DCT-IV seems to work better than other three discrete cosine transforms. Also it has been observed to clearly outperform three of the discrete sine transforms, and to do slightly better than the fourth, as measured by results presented below. The version of DCT-IV implemented in the Wolfram Language is scaled so as to be self-inverting. We actually “center” the image array, by subtracting the mean from each individual value. This removes the DC component from the result.

For many common image types it suffices to retain only a few low frequency components. FCGR images are an exception: due to their fractal nature, it seems that more are needed. We use 30 in each dimension, thus reducing the images to 30×30. We have found this value to give good results across a range of tests, although certainly there is room for further experimentation.

**Fig. 2:**
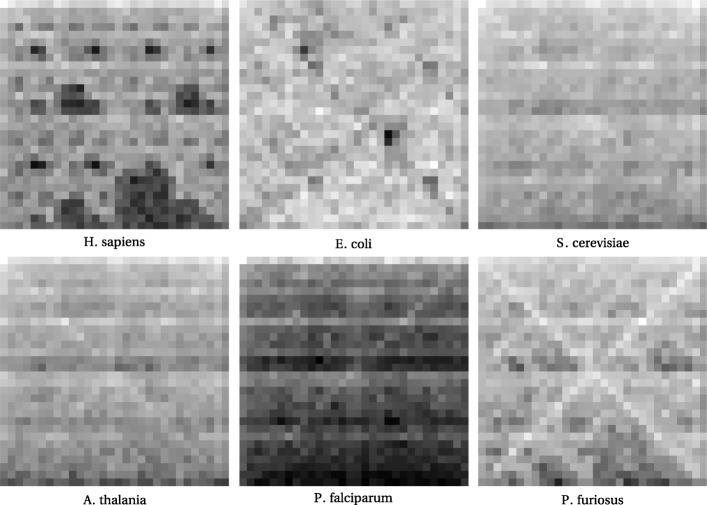
DCT dimension-reduced FCGR images

It might be useful to see an example of this dimension reduction. While a DCT-transformed image is for most viewers not particularly enlightening, an inverse transform brings it back into some semblance of the original form. We show this with the six FCGR images above, using DCTs truncated to 30×30, inverted, and resized to compare to the original images in Fig. 2.

One might ask whether it would be as useful to work with FCGR images created at resolution level of 5, that is, 32 x 32 images. While that can be done, experiments have given consistently better results using the DCT reduction from level 7.

We have also done some experiments using discrete wavelet transforms (DWTs) instead of Fourier Cosine Transforms, as noted in the “Results” section. While the results have not as yet been at the same level as those obtained using the DCT, some have come close. This is an area that could benefit from further exploration. Since we start with images that are 128x128, this step gives a compression factor slightly larger than 18.

#### Singular value decomposition

Once we have a set of matrices resulting from the DCT step, we flatten each and again center by subtracting from each vector its mean value. The purpose of this flattening is to turn each matrix into a vector, and it is done by the common method of appending rows. We stack all these vectors to obtain a single matrix.

The dimension reduction works as follows. We start with a matrix *M*; for our purposes, it is formed from the flattened dimension-reduced vectors resulting from the DCT step. The SVD of *M* gives a trio of matrices (*U,W*,*V*) where *U* and *V* are possibly truncations of orthogonal transform matrices and *W* is a diagonal matrix of singular values. In the full version of SVD, no singular values are removed, and we have the matrix identity *UWV*^*t*^=*M*. Since *V* is an orthogonal matrix (possibly truncated), we thus have *UW*=*MV*. We can do efficient searching as follows. We store the row vectors of the left hand side *UW* in a kd tree [33]. Suppose we want to locate a vector that is in *M*. We multiply on the right by the matrix *V* and the equality of *MV* with *UW* implies that this transformed vector is actually in the kd tree (that is, we have an exact match, modulo tiny machine arithmetic numerical differences that can be ignored for present purposes). If the vector we wish to look up has the same dimension as each row of *M*, but is not itself such a row, we can still find the nearest neighbor efficiently via this same approach: multiply on the right by *V* and search for the nearest neighbor thereof. For modest dimensions (up to tens but not hundreds) a kd tree tends to offer fast lookup.

We reduce dimension by truncating the matrix *W* to retain only *r* singular values, for some modest value *r* (typically in the range of 5-50 for our purposes). This offers several advantages. For one, when *M* is large we reduce considerably on memory consumption. Another is that iterative linear algebra methods can be deployed for modest values of *r*, and these offer advantages over direct methods both in speed and memory consumption. When we reduce dimension in this way the identity *UWV*^*t*^=*M* no longer holds. The left hand side becomes instead the best approximation, in a Euclidean norm measure, of *M* by any matrix of rank *r*. So we now have the approximation *UW*≈*MV*. Our lookup set is comprised of the vectors from the left side of this approximate equality. We store them in a kd tree [4] for purposes of efficient nearest neighbor lookup. These vectors can be used for other purposes as well. We can also compute distances between them for purposes of creating a phylogenetic tree. We can moreover use these vectors to train a neural net, or as training data for other machine learning methods. We show these in the “Results” section.

**Fig. 3:**
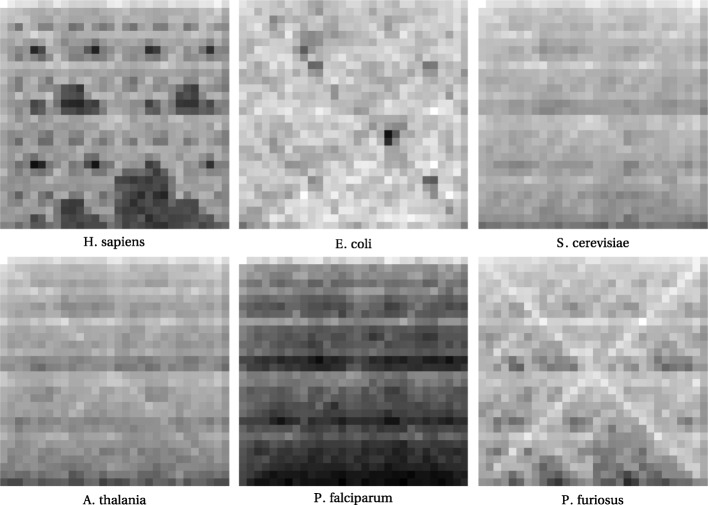
DCT-and-SVD dimension-reduced FCGR images

As with the DCT step, we can show a visual form of this SVD dimension reduction. Multiplying *UWV*^*t*^ gives a matrix with rank *r* but dimension the same as *M*. We show the resulting images in Fig. 3 corresponding to the initial strings for the six genomes seen above, with *r* set to 36. The loss of fidelity appears to be fairly modest.

When using these dimension-reduced vectors the lookup step remains unchanged. In order to find nearest images to a new FCGR image, we again process it with the DCT. We then reduce to dimension *r* by multiplying on the right by the *V* matrix from the SVD step. At this point it has the same dimension as the reference vectors in the kd tree and it is a simple matter to locate the nearest neighbors.

Different applications appear to benefit from retaining different numbers of singular values. The nearest neighbor lookups did well with 20–50, whereas neural net results seemed to do better when we retained around 80 singular values. We used 40 for the SVD step because results did not seem to improve much when we increased it. Experiments showed that use of fewer would result in faster run times and perhaps improved scalability, but at the cost of a modest decrease in accuracy.

A similar method was proposed in [29], albeit without the Fourier dimension reduction step. The authors tested their methods on a dataset comprised of 400 HPV genomes split among 12 genotypes. They attained essentially perfect recognition. The method of this paper likewise attains perfect recognition on their data, over repeated randomized trials. The protocol for these was to take an even split with half of the sequences for training and half for testing. The training data were used for constructing the kd tree. Members of the test set were each ascribed to the class of their corresponding nearest neighbor from the training set.

### Algorithm complexity

We now explain the speed and memory requirements of the several steps. First we establish notation. Nucleotide strings are encoded as arrays of ascii bytes (one for each nucleotide). We will assume we have *m* such strings, each of length *n*. (In some experiments we may have input genome sequences of different length, and we chop these into fixed lengths. For purposes of this subsection, that fixed length will be *n* and the total number of chopped subsequences will be *m*.) Our pixelation level is *k*, the number of Fourier frequencies we retain will be *f*, and the number of singular values we retain will be *s*.

The process of converting a given string into an FCGR image is linear in the string length. As there are 2^2*k*^=4^*k*^ pixels it is also linear in this value, hence is *O*(*n*+4^*k*^). With *m* such strings, the total cost for this step is *O*(*m*(*n*+4^*k*^)).

The DCT is computed using the same underlying method as for the Fast Fourier Transform (FFT). This is *O*(*r* log*r*) where *r* is the total number of elements. As we have 4^*k*^ elements for each image, and *m* images, the total speed complexity is *O*(*mk*4^*k*^). Each image matrix has dimensions *f*×*f* so the memory use is *O*(*mf*^2^). When working with a large data set we typically read in one genome at a time, converting to FCGR image and computing this DCT before processing the next genome string. So this gives the memory footprint for the first two steps.

After flattening these two dimensional DCTs we have a matrix of dimensions *m*×*f*^2^. When retaining the largest *s* singular values, and under the assumptions that *s*<<*f*^2^ and *f*^2^<*m*,the SVD step has time complexity *O*(*mf*^4^). The memory requirement is comparable to the size of the input, so the memory use is *O*(*mf*^2^). At the end of this subsection we indicate two ways to curtail this and also improve speed.

Storage of *m* vectors each with *s* machine double components in a kd tree requires Only *O*(*ms*) memory. The time required to create the tree tends to be *O*(*mlog*(*m*)*s*) and in any case is never a bottleneck for purposes of this methodology.

We now describe algorithmic complexity for lookup. Suppose a genome string to be looked up in our kd tree has *t* base pairs. Conversion to an FCGR image is *O*(*t*+4^*k*^) in algorithmic complexity and also memory use. Taking the DCT is *O*(*k*4^*k*^), and this step produces a vector of length *f*^2^. We use the right multiplier matrix from the SVD step to create the lookup vector. Recall that as we retain *s* singular values and corresponding vectors, this matrix has dimensions *s*×*f*^2^. The matrix multiplication step that gives the lookup vector is therefore *O*(*s*×*f*^2^). The actual lookup in the kd tree takes *O*(*slogm*).

There are ways to improve both the time complexity and memory consumption of the SVD step. One is to take a random subset of the rows to form a smaller matrix, extract the SVD of that new matrix, and use the resulting right multiplier matrix to convert the rows that had been omitted. In effect, this processes the omitted rows in the same manner as we process test vectors. In some experiments this showed only a small loss in overall accuracy. Moreover we can avoid creating the full matrix. We can simply work with a random subset of the genome sequences, create the submatrix, extract the SVD, and process all remaining training sequences one by one (as we can do for the test sequences). The benefit to this approach is that memory usage can be curtailed in situations where the training set is large. This allows to scale to large data sets while using only modest hardware. We illustrate the potential for this approach in a variant of one example. A second approach is to premultiply by the transpose matrix, take the SVD of the resulting square matrix, and use the square roots of the singular values. The right side multiplier matrix is the same, up to row signs, as for the usual SVD, and we do not require the left side multiplier matrix. This approach can be useful for large data sets.

## Materials

### Datasets and experiments

#### Identification of microbial genomes

One data set comes from Martin Swain. It is a curated set of microbial genomes and split into training and test subsets This is described in a prior article by Swain [28] and made available via [37]. The training set has 1053 genome sequences from 565 distinct species. The test set has 650 genomes from 129 distinct species. It turns out that five of those are not represented in the training set. We removed those five species from further consideration; this brought the number of test genomes to 640. We remark that the actual work in [37] used a superset of this data set, with around 50 The taxonomic classifications contained some noise, thus motivating release of the cleaned subset in [37].

We split each genome into nonoverlapping sequences of 20000 bp. We retain from each genome up to 37 such sequences, with starting points approximately equally spaced throughout the genome (for shorter genomes we retain as many subsequences of that length as we can, subject to them not overlapping). The purpose of the experiment was to determine how well the training data could be used to recognize correct genus and species of the test data sequences. We then varied the sequence length by a factor of five in each direction, that is, repeating with sequences of length 4000 and length 100000, in order to gauge the effect of sequence length on quality of results. A variation of this strings together noncontiguous subsequences of 200 bp in order to gauge how well this method will perform if the data is comprised of multiple short reads from a given genome (a similar experiment, albeit with much longer subsequences, is reported in [17]). We also used the majority guess from subsequences of each test genome in order to derive a best guess for genus and species for that entire genome.

A related experiment uses the data set that was also used in [28]. It is available from the web resource reported in [38]. We use the protocol described in [28] to determine species classifications for the test genomes. This provides a means for direct comparison of results between [28] and this paper.

#### Identification of cyprinid genera

The second data set comes from [27]. There are 81 mitochondrial genomes from six genera in the family *Cyprinidae*. This turns out to be a relatively difficult classification: the best of the methods in [27, 30] attains an accuracy of 92.6% (this is of course a good result, but it is somewhat below the scores from other tests presented in that work).

A key point in working with this and the microbial data set is that the method we described occupies a particular “sweet spot”. It is not as fast as the methods used in [27] or [28], but it is far faster than algorithms that use alignment. Moreover it will scale to large sets; the most computationally intensive step is the SVD, and that is mitigated by the fact that the number of columns is limited by the DCT components retained, and we only compute at most a few dozen singular values (this also restricts the memory usage).

#### Construction of phylogenetic trees

We use hierarchical clustering (as built into the Wolfram Language [36]) to construct phylogenetic trees from nucleotide sequences as processed by the method described above. One benchmark data set is comprised of five types of avian Influenza A genomes from 38 samples. It was introduced in [12] and also used in [21, 27]. An optimal tree will separate the five strains. A second example, introduced in [29], uses mitochondrial DNA sequences from 26 species. A third example contains mitochondrial gene sequences from 41 mammalian species. It contains representatives from eight distinct orders, of which all but two have multiple representatives. This was introduced in [21] and used also in [27]. The best results will separate the eight orders, as well as the families for those that families have multiple representatives (e.g. Ursidae, Canidae, and Felidae families in the Carnivora order should be mutually separated).

The accession identifiers for genomes in these three sets are given in the supplement, along with Wolfram Language code for obtaining the nucleotide sequences from GenBank [39]. Finally we use reference sets from [32]. The ascension identifiers for that set are found in the on-line web site corresponding to that work: http://afproject.org/app/. I thank an anonymous reviewer for suggesting this resource as a means for obtaining a quantified comparison with numerous other methods.

#### Inferring host genera from viral phages

A question considered in recent years involves determining likely host bacteria for a given viral genome [1, 8]. One approach is to look for genome similarities such as *k*-mer frequency profiles, partial sequence matches, and so forth. Several reasons have been put forth for why such similarities might exist, among them horizontal transfer of genetic material, and evolutionary pressure to avoid recognition by the host organism; see [1, 8] for details. We work with a data set comprised of 820 viral sequences and 2699 possible bacterial hosts. This set was introduced in [8] and the authors have kindly made it available for reference benchmarking.

## Results

### Microbial genomes

The first test of this method was on fragments of length 20000 bp from the training and test sets of microbial species in [37]. We deduce species of a test specimen from the species of the nearest (in Euclidean distance) training specimen vector. We also check for the 20 nearest training specimens, as this can be useful for determining a candidate pool in cases where the first guess might be incorrect. We used FCGR images that are 128x128, retaining a 30×30 matrix of low frequency components from the DCT step, and vectors of length 40 from the SVD step. The correct genus was determined for 91.5% of all fragments, with 97.4% having the correct one among the top 20 neighbors. The correct species was determined in 82.9% of the cases and 95.5% had the correct species in the top 20%. There are 23384 training sequences and 14339 test sequences. For timings, it took 16 min to read in and process all training and test genomes through the DCT step, 7 s to do the SVD step on the training vectors, and 3 s to use the resulting right multiplier matrix to put the test vectors into the correct dimension and then compute the nearest neighbors for all the test vectors.

When we take the majority guess from all subsequences of each of the 640 test genomes, the correct genus is determined for 619 (96.7%) and the correct species for 576 (90.0%).

In a variation of this experiment we retain 80 rather than 40 singular values in the SVD step, and we use the training vectors to train a simple neural net (comprised of a linear layer, a ramp function a hyperbolic tangent, a second linear layer to reduce to the number of classes, and a soft-max layer). The neural net took 18 min to train, and 3 s to run on the 14339 test vectors. The outcome had 85.9% correct species recognition and 93.9% correct genus recognition.

It is of interest to note that individual genomes exhibit considerable self-similarity. When we check the eight nearest neighbors in the training set to each fragment therein (so obviously the first hit is the fragment itself), we find that 92.0% have a nearest neighbor in the same genome. Recall that these were taken from nonoverlapping subsequences, and moreover for those genomes of sufficient (ength (the majority) the fragments were separated by gaps.

We repeated the fragment recognition test using nucleotide segments of 4000 bp. The genus was correctly recognized for 71.0% of the fragments, with the correct genus among the nearest 20 neighbors for 94.1%. The correct species was identified for 61.0%, with the correct one among the nearest 20 neighbors for 90.2%. With the neural net approach the correct species was identified for 70.2% and the correct genus for 78.5%.

We also did this experiment using nucleotide segments of 100000 bp. Here the genus was correct for 95.5% of the test examples, with 97.4% having the correct one in the nearest 20 neighbors. The species was correct for 88.3%, with the actual species among the 20 closest neighbors for 96.1%. A trained neural net did slightly worse for species identification, getting 86.9% correct. The genus level was about the same as for the nearest neighbor approach, getting 95.8% correct. As an indication of what might be done with still longer sequences, we ran the nearest neighbor lookup using training and test sequences of 500000 bp. The species recognition rate here is 90.7%, with 97.2% having the correct species among the 20 closest neighbors. The corresponding numbers for genus recognition are 96.7% and 98.0%.

Table 1 shows timings vs. chunk size. One will note that increasing chunk size means longer processing time for the DCT step, with the SVD and lookup steps dropping once we hit a chunk size that causes the number of training and test vectors to decrease.

**Table 1:** Timing vs chunk size

chunk size	read+FCGR+DCT (min)	SVD+kd-tree (sec)	lookup (sec)
4000	5.5	7	4.6
20000	16	7	3
100000	71.8	7	1.8
500000	93	1.7	0.2

Table 2 summarizes accuracy vs. chunk size.

**Table 2:** Accuracy vs chunk size

chunk size	% genus	% genus nearby	% species	% specied nearby
4000	71.0	94.1	61.0	90.2
20000	91.5	97.4	82.9	95.5
100000	95.5	97.4	88.3	96.1
500000	96.7	98.0	90.7	97.1

Another experiment on this data set was as follows. We treat the training set as usual, using fragments of 20000 bp. We took discontiguous nucleotide fragments of 200 bp from each genome in the test set. We then “stitched together” longer fragments by partitioning each genome’s fragments into groups of 20 (thus forming a matrix) and then transposing the matrix. We still manage to recognize the correct genus for 91.7%, and we get the correct species for 82.8%. The implication is that this method might be of use even when test genome fragments are relatively short (a few hundred bp, say), provided there are multiple fragments for each genome so that one can get a total length of several thousand or more bp.

We remark that the neural net method for recognition will not scale so well as the basic nearest neighbor approach. The training requires considerably more time and memory. That said, we note that we did not try this on a large machine, and we did not make any effort to speed the training via parallelization or use of GPUs.

The data set used above was kindly made available to me, and is now publicly available as [37]. It is a cleaned and condensed version of what was originally used in [28]. In order to have a fair comparison to results presented there, variants of the preceding experiments were performed using the data set from [38] since this was the data set actually used in [28]. This set has 1551 training sequences and approximately 1000 test genomes (due to differences in download dates and possibly also in string processing used for determining taxonomy, there are 1008 test sequences in this paper whereas only 977 were used [28]). We use 100 non-overlapping substrings of 10000 bp from each training sequence (or as many as are available, if fewer than 100) to create the lookup tree. We use four subsequences of that length from each test sequence, since this was the number used in [28]. The sequence with the nearest match in the lookup tree is then used to classify the test sequence (again following the protocol from [28]). The correct species is identified for 64.2% of the sequences. The best method in [28] achieved a correct recognition rate of 46%. As in that reference, this experiment was repeated with subsequences of 100000 bp (where we now use up to 50 subsequences per training sequence). The species is now correctly identified for identified 74.1% of the test sequences. the best method in [28] found the correct identification for just over 67% ([28] also reports that the top subsequence BLAST hit correctly identified the species for roughly 83% of the test sequences). Our method performed substantially better at the genus level in these experiments, identifying the correct one for 88.3% when using 10000 bp subsequences, and with 95.1% correct for the 100000 bp subsequences. The current results compare well with the BLAST results reported in [28] for subsequences of 100000 bp. That gets correct species for 83% and correct genus for 91%. It should be mentioned that there is a tradeoff insofar as the methods used in [28] (excluding BLAST) involve somewhat faster processing than the method in this work.

Returning to the smaller data set from [37], we can offer some modest comparison results. Martin Swain ran and reported his protocol at several subsequence lengths (Swain, private communication). We compare at two fragment lengths that are relatively close to those shown earlier. Recall that the test set is comprised of four subsequences of given length from each of the test sequences. The one that is closest to a training fragment determines the guess for that test sequence. For subsequences of 25600 bp the best Swain method obtains 79.5% correct classification at the species level. Our method has 87.0% correct. When subsequences of 102400 bp are used, the best Swain result has 85.5% correctly classified. In comparison, we get 92.6% correct.

### Cyprinids

This data set contains genomes from six genera in the *Cyprinidae* family. For each genus there are between 10 and 19 individual genomes, with 81 in total. Every genome has about 17000 bp. We do not work with subsequences but rather use the entire nucleotide sequence from each. As in the microbe set, we create FGCR images at a pixelation level of 7, we keep a 30×30 matrix at the DCT step, and we reduce to dimension 40 at the SVD step.

From each genus we take 75% of the individual genomes for training and the rest for testing (we round down for the training set for genera with counts not divisible by 4). We average the results over 1000 such randomized trials.

Processing time is 1.7 s to convert genome sequences to DCT vectors (thus this includes the FCGR step). It takes 3.3 s to run 1000 trials; each involves computing the SVD matrix for 3/4 of the DCT vectors, making the kd tree, converting the remaining 1/4 vectors for lookup and performing the lookups.

The result is that 96.5% of the test samples are correctly identified. Also 99.0% are accounted for by the first two guesses. The best result from [27] has 92.6% correctly identified. We remark that again there is a clear tradeoff, insofar as our method, while more accurate, requires more processing time.

This data set is reasonably small so it can be used to show the importance of the first dimension reduction (the DCT step). Recall that reducing dimension from 128×128 to 30×30 implies a size reduction factor of 18.2. When we omit this step, the 1000 random trials (with timing dominated by the repeated SVD step) goes from 3.3 to 65.4 s, for a commensurate slowdown factor of 19.8. It is also of interest that the quality of result declined modestly, with 95.3 appearing in the two top guesses. A possible explanation is that in retaining only lower frequency components, the DCT effectively blurs image details that might detract from the recognition task. Stated differently, it might be removing what amounts to noise for purposes of FCGR image comparison

A related experiment involves seven phyla from the Animalia family used in [27]. In contrast to the Cyprinidae data set, this one is quite unbalanced, with phyla containing 4367, 1572, 403, 127, 100, 60, and 40 genomes in descending order of size. The best method in [27] attained correct classification of 96.2%. Our method, with the same parameter settings as above (but using 100 randomized trials since the data set was so much larger) correctly classified 98.18%.

This data set contains 6673 sequences. Timing for this experiment was dominated by the time required to read in the genome sequences from a github site; this and the processing through the DCT step took 986 s. The 100 random trials took 138 s; each such trial performed the SVD step on a 5004×900 matrix and did processing and lookup on 1669 vectors of length 900.

### Phylogenetic trees

In the test sets below we again retain the 30×30 matrix of lowest frequency DCT components. When possible we reduce to dimension 40 in the SVD step; those sets with fewer than 40 members do not get reduced in this step. Some experiments indicate that the cosine distance does a slightly better job than the Manhattan distance for purposes of grouping by taxonomy, so we show trees produced with that distance measure.

#### Influenza a data set

**Fig. 4:**
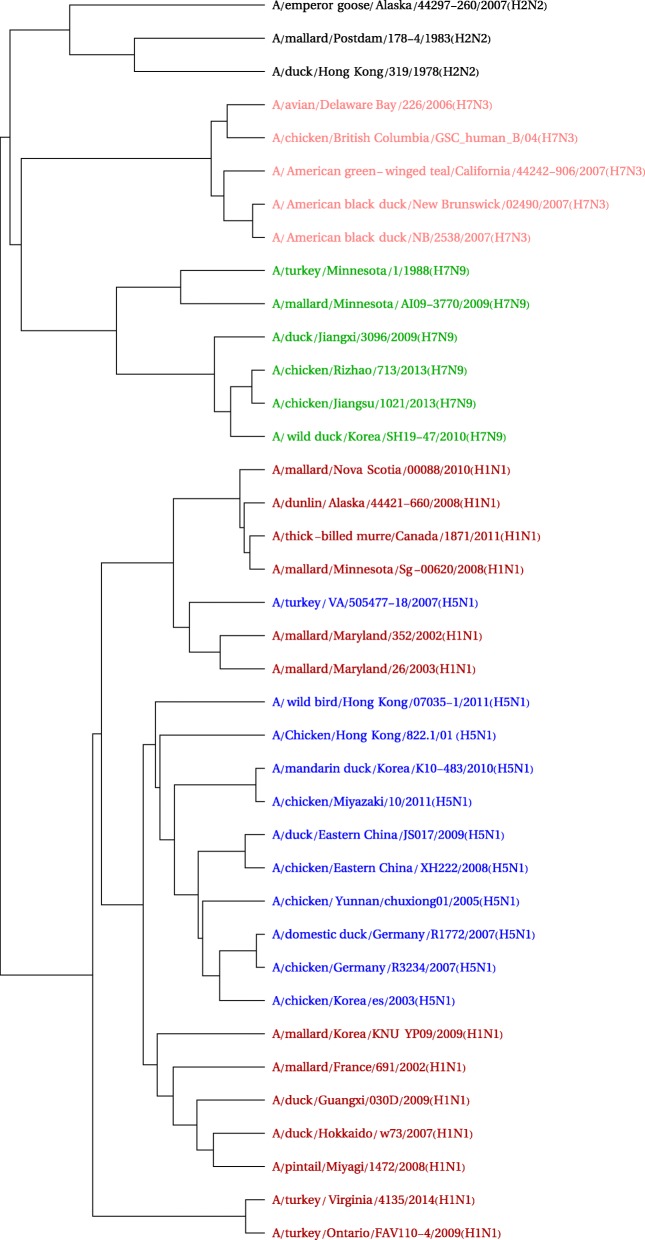
Influenza A phylogenetic tree

The influenza A test set grouping is given in Fig. 4, using the same color scheme as was found in [27] (and similar is used in [21]. The 38 sequences come from five strains, each with a separate color. It shows the same issue that is reported in those references for the alignment-based CLUSTAL Omega program [40]: the A/turkey/VA/505477-18/2007(H5N1) is placed in the H1N1 group (though this misplacement does not happen for the methods developed in [21, 27]). It can be seen that, while the remaining H5N1 viruses appear in the middle of the H1N1 group, this is in part due to a vagary of graph layout: the H5N1 cases are in fact grouped together and (with the noted exception) separated from the H1N1 group. The distances between the two “separated” parts of the H1N1 viruses do indicate a modest weakness in the clustering though (and it is also seen in the CLUSTAL omega grouping) [21, 27]. It is perhaps notable that, as with the CLUSTAL Omega result from those references, groupings show geographical affinities. This is the case, for example, with the separated subgroups of the H1N1 strain. This type of affinity makes sense if substrains tend to be geographically clustered. Curiously, the errant H5N1 is the only sample from that strain that came from North America, as the others are from east Asia and Germany. It is from Virginia and is placed in proximity to two H1N1 samples from Maryland.

The full processing time, excluding the time needed to download sequences from GenBank [39], is 0.1 s. It took 47.5 s to obtain the 38 sequences from the GenBank site.

**Fig. 5:**
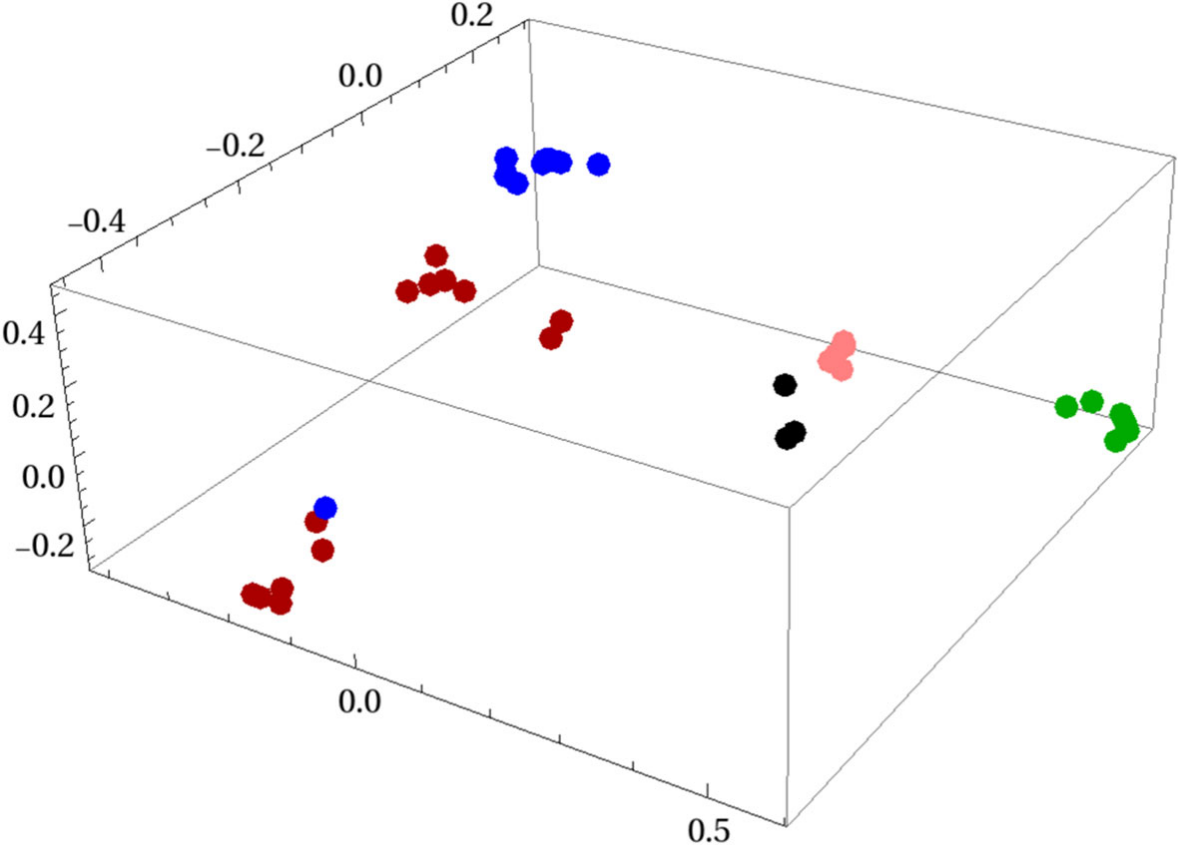
Influenza A 3D MDS plot

Figure 5 shows a Multidimensional Scaling (MDS) plot in three dimensions. This MDS scaling is created from the distance matrix between vectors that emerge from the FCGR-DCT-SVD processing. One can see the lone H5N1 sequence appearing amidst several H1N1 genomes. It also indicates a separation of the H1N1 sequences into two distinct subsets.

**Fig. 6:**
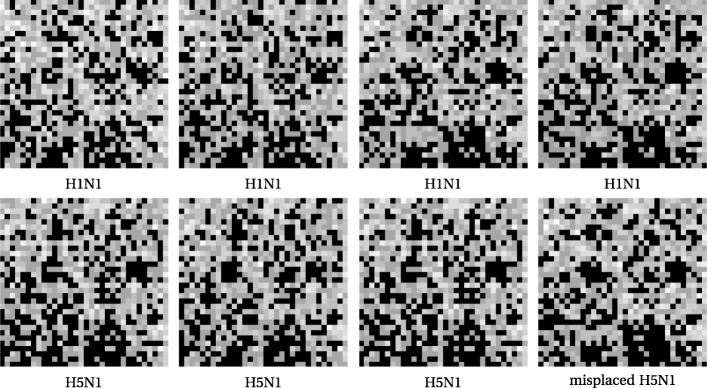
Low resolution FCGRs for four H1N1 and four H5N1

**Fig. 7:**
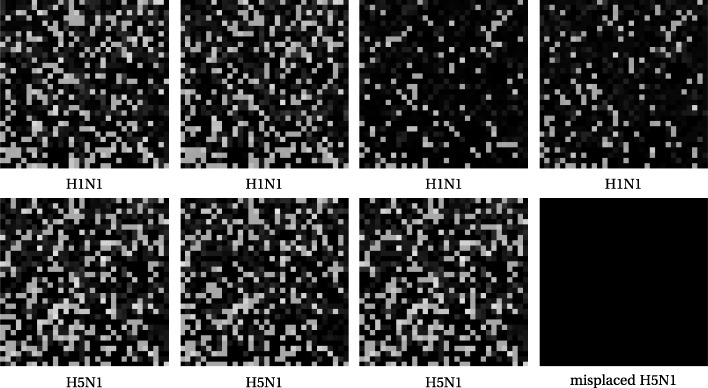
Image differences with misplaced H5N1

One might ask whether the proximity of the one H5N1 sequence is a vagary of reduction to three dimensions, or an artifact of the lossy steps of DCT and SVD. In fact it is neither. We illustrate this with Figs. 6 and 7. The first row of Fig. 6 is comprised of FCGR images, at pixelation level of 5, of four H1N1 sequences. The two on the left are from specimens in Asia and the two on the right are from Maryland, USA. The second row has the first three and last of the H5N1 sequences; it is that last that is the outlier in the tree and MDS plot.

While it is by no means obvious to the eye that the image for the misplaced sequence is "closer" to the H1N1 family, we can see this by taking differences of each image with that misplaced one. Recalling that black pixels come from values of zero, it is now fairly visible in Fig. 7 that the errant H5N1 on the bottom right is closest to the two H1N1 FCGR images on the upper right.

#### Mitochondrial data set

**Fig. 8:**
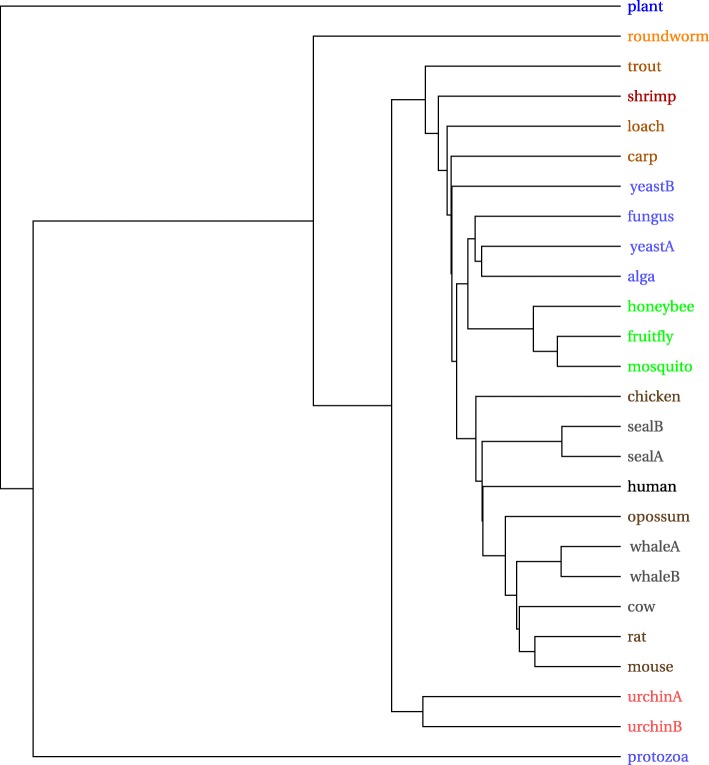
Eukaryotic mitochondrial phylogenetic tree

The mitochondrial DNA tree in Fig. 8 comes from longer genomes but still took under a second to create, starting with the nucleotide sequences. It bears some similarity to the Euclidean and image distance trees in Figure 2 of [30] (one of which also places chickens and humans as neighbors). The bottleneck, again, was in obtaining the 26 genome sequences from GenBank; this required 30 s.

**Fig. 9:**
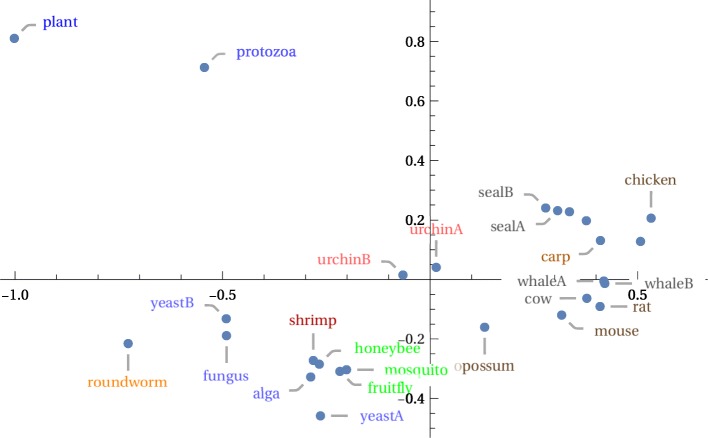
Eukaryotic mitochondrial 2D MDS plot

An MDS plot in two dimensions appears to be consistent with the phylogenetic tree in Fig. 9. It also shows that the plant and protozoa, while far apart, are mutually closer to one another than they are to other species. The tree, due to artifact of the drawing choices, obscures this fact. The MDS plot also has a better placement of the shrimp.

#### Mammalian mitochondrial data set

**Fig. 10:**
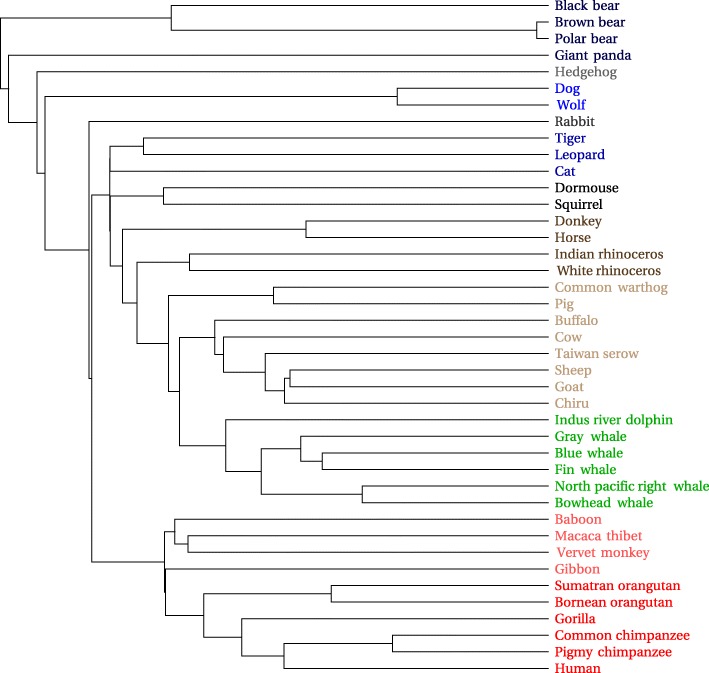
Mammalian mitochondrial phylogenetic tree

The mammalian mitochondrial DNA tree in Fig. 10 is similar to that in [21]. Total processing time was 0.9 s, with 45 s needed to download the 41 sequences from GenBank.

**Fig. 11:**
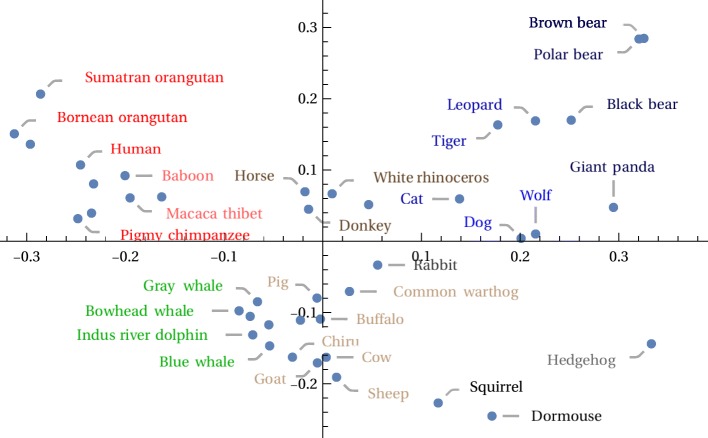
Mammalian mitochondrial 2D MDS plot

Clustering appears to be slightly better for the Carnivora order than that shown in [21], insofar as Ursidae are all grouped together here. Moreover greater apes (family Hominidae), lesser ape (family Hylobatidae), and monkeys (family Cercopithecidae) are mutually separated. The hedgehog and rabbit, however, appear within the Carnivora. It is not obvious to what extent this is an artifact of the drawing layout (they could both have been rotated downward and would then appear on the bottom) vs. how much it indicates an error in the actual tree. A two dimensional MDS plot in Fig. 11, created from the same vectors serves to clarify this, as it shows the rodents, hedgehog, and even the rabbit to be apart from the Carnivora. A three dimensional plot, which supports manual rotations, would show the desired separations even more clearly.

Finally we indicate how this method fares on a benchmark computed using several other methodologies. We use data sets from [32]. There are 61 reference methods shown at the web site http://afproject.org/app/benchmark/genome/std/assembled/plants/dataset/. They all differ from the "gold standard" reference tree. For the plant data set the Robinson-Foulds (RF) distances range from 2 to 20. The method of this paper had RF distance of 14. A data set comprised of mitochondrial fish DNA from that same web resource had RF distances ranging from 2 to 44, with our method having RF distance 20.

The examples indicate that this method is useful insofar as it is both fast and able to produce results comparable to prior work. That said, it is not perfect, and perhaps would benefit from synthesis with other methods.

### Predicting viral hosts from viral and bacterial sequences

The data set from [8] contains 820 viral phages sequences and 2699 bacterial sequences. Among the latter are host bacteria species for each of the former. In the Additional file 1 accompanying [8] the authors provide correctness percentages for species, genus,..., phylum recognition using variants of five general approaches. A total of fourteen tables are shown. The genus recognition percentages range from 15 to 62.3. Two of the fourteen exceed 50%. One, based on exact matches between genomes, attains 50.4% correct genus recognition. Another, using blastn search to compare genetic homology, achieves a correct recognition rate of 62.3%. The exact matching method gives 2.4 guesses on average per phage genome, with a phage being ascribed as correctly matched if any of the guesses hit the correct host genus. The genetic homology method used, on average, only 1.4 guesses per genome, and clearly the fewer guesses required, the more powerful the method.

We use the proximity measure described in prior sections to ascribe possible host genera to the phages. The specific protocol followed used full viral genomes, and chunks of length 20000 bp for the bacterial genomes (allowing for smaller in the few cases where the bacterial genome did not meet this size threshold). As in most prior examples, we created FCGR images of size 128x128 (pixelation level 7). The DCT step retained the 30×30 matrix of lowest frequency components. The flattened submatrices were processed in the SVD step retaining the 40 largest singular values, and a kd tree for lookup was produced.

We then find, for each viral genome vector, the 28 nearest bacterial chunks and their corresponding genera. We retain all genera that appear at least twice in a given list in order to insist that there be at least a modest level of consensus. We chose 28 initial neighbors because, coupled with the two-or-more consensus requirement, this gave very close to an average of three genus predictions per viral genome. We regard as correct any phage that has the correct genus among its retained predictions. With these parameters, 56.0% of the viral genomes are ascribed their correct genera. If we only take the 14 nearest neighbors then the average number of genera that appear at least twice drops to two, and correctness drops to 51.7%. With 44 nearest neighbors and consensus of two or more, there are on average four predictions per phage, and 61.0% have the correct genus among their predicted genera. Based on these values it would be reasonable to state that the method performs somewhere near the middle of the two most successful prediction methods described in [8].

We did similar computations at the Order, Family, and Species levels, in all cases using as many neighbors as would have the average guess count closest to three. We show a table with our results along with the first 13 (of 14) method results from the Additional file 1 accompanying [8] (the last had to be omitted because the next-to-last was inadvertently repeated in that reference). Full details of their methods are found in the reference; we use their nomenclature in Table 3.

**Table 3:** Phage classifier results

Method	*#* guesses avg	% order	% family	% genus	% species
FCGR-to-vector	3.00	78.90	67.30	56.00	40.00
Coabundance	1.74	27.20	22.20	15.90	12.28
homology by blastn	1.40	81.46	73.90	62.32	45
homology by blastx	1.61	66.95	56.10	48.83	32.68
CRISPR method 1	3.93	36.46	31.10	1.22	15.49
CRISPR method 2	1.34	37.44	26.95	26.95	21.71
Exact matches	2.42	61.22	57.32	50.37	40.49
3-mer frequency	1.49	36.71	29.15	21.59	8.17
4-ramer frequency	1.40	37.20	32.80	24.80	9.76
5-mer frequency	1.39	42.32	36.46	28.78	12.32
5-mer frequency	1.29	45.61	40.12	31.34	12.93
7-mer frequency	1.28	46.10	41.10	32.93	14.51
8-mer frequency	1.33	46.71	42.56	34.63	17.07
Codon similarity	17.00	42.07	19.76	15.24	10.37

We now provide processing time details. It takes 20 min to retrieve the 820 viral phage genomes from GenBank and process them through the FCGR and DCT steps. It takes 5 h to obtain the (substantially longer) 2699 bacterial genomes from GenBank, partition into segments of 20000 bp (discarding the remaining subsequence at the end), and further process these through the FCGR and DCT steps. The genomes in this set have, on average, 3.2 million bp, and in total there are 429642 subsequences of 20000 bp. These comprise our training set. It takes 11 min to further process this set through the SVD and kd tree steps. The SVD step, however, requires the most memory, using approximately 9 Gb (the input matrix is itself 3.24 Gb). Preprocessing and lookup of the 820 phage sequences takes 12 s. So the substantial bottleneck is the tandem of obtaining the sequences from GenBank, creating from each an FCGR image array and running a DCT thereon.

A variant of this experiment was run in order to assess a speed/memory improvement. Every fourth sequence was extracted from the DCTs of the bacterial genomes in the training set and run them through the SVD step. We use the right multiplier matrix to convert the remaining 3/4 of the training set to the correct dimension, and then merge that with the initial 1/4 to create a kd tree from the full training set. This makes the SVD more efficient by a factor of four. The quality of the lookup for the taxonomy level we tested, genera, dropped from 56.0% to 54.6%.

## Conclusions

We have shown a method of translating DNA sequences into vectors of modest size, in a way that allows for distance-based comparisons. Sequences are first transformed to images using the Frequency Chaos Game Representation. The Discrete Cosine Transform is used to reduce dimensions, with the low frequency components retained. These smaller matrices are flattened into vectors, and the Singular Values Decomposition is used to further reduce dimension. These vectors can now be used for hierarchical clustering to produce a phylogenetic tree, or they can be used in a kd tree for efficient nearest neighbor lookup. The steps are all straightforward and computationally well behaved; the most strenuous step, in terms of memory consumption, is in computing the SVD. Several experiments indicate that this approach scales well and moreover imposes no requirement that nucleotide sequences all have a common length.

We used this approach in several nontrivial classification tests. Results at the species and genus level compare quite favorably with prior literature. Also of importance is that the methodology is relatively cheap from a computational perspective. Since they are fairly short, the vectors produced by this dimension reduction could serve as genetic “fingerprints”, suitable for (at a minimum) coarse-grained genetic lookup tasks. For example, one might use them to assess taxonomic order, family, or perhaps even genus, and then resort to slower but more powerful comparison methods to drill deeper. Another advantage is that these can use short sequence reads strung together and still obtain reasonable results, as was seen in one variation from the experiments using a microbial genome data set.

We performed substantial experiments to determine good hyperparameter values (e.g. pixelation level, Fourier frequency cut-off, singular values, and cut-off). But this could benefit from further study and indeed the optimal values may very with the type of classification. One important point, however, is that all tests indicate these need not be terribly large. For example, results do not seem to improve much with pixelation levels larger than 7, retention of more than 30 Fourier frequencies, or more than the 40 largest singular values. This is important insofar as it places approximate upper bounds on both algorithmic complexity and memory requirements.

## Availability and requirements

**Project name**: GenSeqCompbyFCGRandSigProc

**Project home page**: Not applicable

**Operating system(s)**: Platform independent

**Programming language**: Mathematica version 11.1 or higher

**Other requirements**: None

**License**: Creative Commons Attribution ShareaAlike (CC BY-SA)

## Supplementary information

**Additional file 1** Wolfram Language code for all computations herein.

## Data Availability

Links to all datasets, as well as all code used to obtain and analyze them, are included in the Additional file 1. This includes NCBI accession numbers and code to download corresponding genome sequences.

## Abbreviations

BLAST: Basic local alignment search tool
bp: Base pairs
CGR: Chaos game representation
DCT: Discrete cosine transform
DWT: Discrete wavelet transform
FCGR: Frequency chaos game representation
HPV: Human Papillomavirus
KNN: K-nearest neighbors
SVD: Singular values decomposition

## References

[1] Ahlgren NA, Ren J, Lu YY, Fuhrman JA, Sun F (2017). Alignment-free d$_{2}^{*}$ oligonucleotide frequency dissimilarity measure improves prediction of hosts from metagenomically-derived viral sequences. Nucleac Acids Res.

[2] Almeida JS, Carriço JA, Maretzek A, Noble PA, Fletcher M (2001). Analysis of genomic sequences by Chaos Game Representation. Bioinformatics.

[3] Anastassiou D (2000). Frequency-domain analysis of biomolecular sequences. Bioinformatics.

[4] Berger B, Peng J, Singh M (2013). Computational solutions for omics data. Nat Rev Genet.

[5] Borrayo E, Mendizabal-Ruiz EG, Vélez-Pérez H, Romo-Vázquez R, Mendizabal AP, Morales JA (2014). Genomic signal processing methods for computation of alignment-free distances. PLoS ONE.

[6] Cristea DP (2003). Large scale features in DNA genomic signals. Signal Process.

[7] Deschavanne PJ, Giron A, Vilain J, Fagot G, Fertil B (1999). Genomic signature: characterization and classification of species assessed by Chaos Game Representation of sequences. Mol Biol Evol.

[8] Edwards RA, McNair K, Faust K, Raes J, Dutilh BE (2016). Computational approaches to predict bacteriophage-host relationships. FEMS Microbiol Rev.

[9] Farkaš T, Sitarčík J, Brejová B, Lucká M (2019). Swspm: a novel alignment-free comparison method based on signal processing. Evol Bioinforma.

[10] Hamori E, Ruskin J (1983). H curves, a novel method of representation of nucleotide series especially suited for long DNA sequences. J Biol Chem.

[11] Haubold B (2013). Alignment-free phylogenetics and population genetics. Brief Bioinforma.

[12] Hoang T, Yin C, Yau SS-T (2016). Numerical encoding of DNA sequences by chaos game representation with application in similarity comparison. Genomics.

[13] Hou W, Pan Q, He M (2014). A novel representation of DNA sequence based on CMI coding. Phys A Stat Mech Appl.

[14] Jeffrey HJ (1990). Chaos game representation of gene structure. Nucleic Acids Res.

[15] Joseph J, Sasikumar R (2006). Chaos game representation for comparison of whole genomes. BMC Bioinformatics.

[16] Karamichalis R, Kari L, Konstantinidis S, Kopecki S (2015). An investigation into inter- and intragenomic variations of graphic genomic signatures. BMC Bioinformatics.

[17] Karamichalis R, Kari L, Konstantinidis S, Kopecki S, Solis-Reyes S (2016). Additive methods for genomic signatures. BMC Bioinformatics.

[18] Kubicova V, Provaznik I (2016). Use of whole genome DNA spectrograms in bacterial classification. Comput Biol Med.

[19] Kuksa PP, Pavlovic V (2009). Efficient alignment-free DNA barcode analytics. BMC Bioinformatics.

[20] Li CX, Fei W, Zhao Y, Vishwanath T (2016). Novel graphical representation and numerical characterization of DNA sequences. Appl Sci.

[21] Li Y, He L, He RL, Yau SS-T (2017). A novel fast vector method for genetic sequence comparison. Sci Rep.

[22] Liao B, Ding K (2006). A 3D graphical representation of DNA sequences and its application. Theor Comput Sci.

[23] Loh P, Baym M, Berger B (2012). Compressive genomics. Nat Biotechnol.

[24] Mendizabal-Ruiz G, Román-Godínez I, Torres-Ramos S, Salido-Ruiz RA, Morales JA (2017). On DNA numerical representations for genomic similarity computation. PLoS ONE.

[25] Pei S, Dong W, Chen X, He RL, Yau SS-T (2019). Fast and accurate genome comparison using genome images: the extended natural vector method. Mol Phylogenet Evol.

[26] Pei S, Dong R, He RL, Yau SS-T (2019). Large-scale genome comparison based on cumulative Fourier power and phase spectra: central moment and covariance vector. Comput Struct Biotechnol J.

[27] Randhawa GS, Hill KA, Kari L (2019). ML-DSP: machine learning with digital signal processing for ultrafast, accurate, and scalable genome classification at all taxonomic levels. BMC Genomics.

[28] Swain Martin T. (2013). Fast Comparison of Microbial Genomes Using the Chaos Games Representation for Metagenomic Applications. Procedia Computer Science.

[29] Tanchotsrinon W, Lursinsap C, Poovorawan Y (2015). A high performance prediction of HPV genotypes by Chaos Game Representation and singular value decomposition. BMC Bioinformatics.

[30] Wang Y, Hill K, Singh S, Kari L (2005). The spectrum of genomic signatures: from dinucleotides to Chaos Game Representation. Gene.

[31] Xie G, Mo Z (2011). Three 3D graphical representations of DNA primary sequences based on the classifications of DNA bases and their applications. J Theor Biol.

[32] Zielezinski A, Vinga S, Almeida J, Karlowski WM (2017). Alignment-free sequence comparison: benefits, applications, and tools. Genome Biol.

[33] Bentley JL (1975). Multidimensional binary search trees used for associative searching. Communun ACM.

[34] Zhang D, Ding D, Li J, Liu Q. A PCA-based face recognition method by applying fast Fourier transform in preprocessing. In: 3rd International Conference on Multimedia Technology (ICMT 2013): 2013. p. 1155–62. 10.2991/icmt-13.2013.141.

[35] Lichtblau D. Linking Fourier and PCA methods for image look-up. In: 2016 18th International Symposium on Symbolic and Numeric Algorithms for Scientific Computing (SYNASC). IEEE: 2016. p. 105–10. 10.1109/SYNASC.2016.028.

[36] Wolfram Research. Mathematica 12.0. 2019.

[37] Swain MT. Microbial genome sequences and taxonomic information based on the Genometa 2012 data set. 2019. 10.20391/e6974906-f30f-4976-90fb-ea1679eedef0.

[38] Davenport C, Neugebauer J, Beckmann N, Friedrich B, Kameri B, Kokott S, Paetow M, Siekmann B, Wieding-Drewes M, Wienhöfer M, Wolf S, Tümmler B, Ahlers V, Sprengel F (2012). Genometa - a fast and accurate classifier for short metagenomic shotgun reads. PLoS ONE.

[39] Benson DA, Cavanaugh M, Clark K, Karsch-Mizrachi I, Lipman DJ, Ostell J, Sayers EW (2016). GenBank. Nucleic Acids Res.

[40] Sievers F, Wilm A, Dineen D, Gibson TJ, Karplus K, Li W, Lopez R, McWilliam H, Remmert M, Söding J, Thompson JD, Higgins DG (2011). Fast, scalable generation of high-quality protein multiple sequence alignments using Clustal Omega. Mol Syst Biol.

