# Supplementary material for: Alignment-free genomic sequence comparison using FCGR and signal processing
Source: BMC Bioinformatics. 2019 Dec 30;20:742. doi: 10.1186/s12859-019-3330-3 (PMC6937637; doi:10.1186/s12859-019-3330-3)
Supplement: Supplementary file 1 — Additional file 1 Wolfram Language code for all computations herein. [file 12859_2019_3330_MOESM1_ESM.pdf]

Supplementary material for “Alignment-free genomic sequence comparison using FCGR and signal processing”

FCGR code for producing images of dimension 2^7 $x\,2^7$ from each sequence of length 150,000.

In[ ]:=
```
srules = {"U" → "T", Except[Characters["ACGT"]] → ""};
seglen = 20 000; (* 10000, 20000, 50000, 100000 *)
chars = {"A", "T", "G", "C"};
dim = 7; (* 6-8 *)
freq = 30;(* 20-40 *)
keep = 40;(* 24-64 *)
replace = Dispatch[Thread[chars -> {{0, 0}, {0, 1}, {1, 1}, {1, 0}}]];
```

In[ ]:=
```
makePositionsC = Compile[{{shifts, _Integer, 2}, {k, _Integer}},
    Module[{posns},
      posns =
       FoldList[IntegerPart[(#1+#2) / 2.] &, {2^k / 2, 2^k / 2}, (2.^k) * shifts];
      Rest[1 + posns]
    ], RuntimeOptions → "Speed", CompilationTarget → "C"];

FCGR[chars_String, k_] := Module[
  {shifts, posns, newposns},
  shifts = Characters[StringReplace[ToUpperCase[chars], srules]] /. replace;
  newposns = Round[makePositionsC[shifts, k]];
  (*Normal[*)SparseArray[Apply[Rule, Tally[newposns], {1}], {2^k,  2^k}](*]*)
 ]
```

Create FCGR image and take Fourier trig transform.

In[ ]:=
```
processNucleotideString[chars_, dim_, freq_] := Module[
  {fcgr = FCGR[chars, dim], ftt},
  fcgr = (fcgr / N[Max[fcgr]])^(1 / 5);
  ftt = Flatten[FourierDCT[fcgr - Mean[Flatten[fcgr]], 4][[1 ;; freq, 1 ;; freq]]];
  ftt - Mean[ftt]
 ]
```

Create a lookup table based on the SVD decomposition

In[ ]:=
```
imageKNN[ivecs_, vals_, keep_] :=
 Module[
  {uu, ww, vv, udotw, norms},
  {uu, ww, vv} =
   SingularValueDecomposition[ivecs, keep];
  udotw = uu.ww;
  norms = Map[Sqrt[#.#] &, udotw];
  udotw = udotw / norms;
  udotw = Join[udotw, Transpose[{Log[norms]}], 2];
  {Nearest[udotw → vals, Method → "KDTree"], vv}]
```

Process test vectors so they can be looked up.

In[ ]:=
```
processTestInput[ivecs_, vv_] :=
 Module[
  {tdotv, norms},
  tdotv = ivecs.vv;
  norms = Map[Sqrt[#.#] &, tdotv];
  tdotv = tdotv / norms;
  tdotv = Join[tdotv, Transpose[{Log[norms]}], 2];
  tdotv]
```

Do SVD but do not create a lookup table.

In[ ]:=
```
trainImageProcess[ivecs_, keep_] :=
 Module[
  {uu, ww, vv, udotw, norms},
  {uu, ww, vv} =
   SingularValueDecomposition[ivecs, Min[keep, Length[ivecs]]];
  udotw = uu.ww;
  norms = Map[Sqrt[#.#] &, udotw];
  udotw = udotw / norms;
  udotw = Join[udotw, Transpose[{Log[norms]}], 2];
  {udotw, vv}]
testImageProcess[ivecs_, vv_] :=
 Module[
  {tdotv, norms},
  tdotv = ivecs.vv;
  norms = Map[Sqrt[#.#] &, tdotv];
  tdotv = tdotv / norms;
  tdotv = Join[tdotv, Transpose[{Log[norms]}], 2];
  tdotv]
```

In[ ]:=
```
trainImageProcessB[ivecs_, keep_] :=
 Module[
  {uu, ww, vv, udotw, norms},
  {uu, ww, vv} =
   SingularValueDecomposition[ivecs, Min[keep, Length[ivecs]]];
  udotw = uu.ww;
  norms = Map[Sqrt[#.#] &, udotw];
  udotw = udotw / norms;
  udotw = Join[udotw, Transpose[{Log[norms]}], 2];
  udotw]
```

In[ ]:=
```
Needs["HierarchicalClustering`"];
```

Scrape the NCBI for genome sequences.

In[ ]:=
```
link = "https://www.ncbi.nlm.nih.gov/nuccore/";
getfasta[seqref_] :=
 Module[{}, StringCases[Import[link <> seqref <> ".1?report=fasta", "Text"],
    Shortest["<meta name=\"ncbi_uidlist\" content=\"" ~~ z___ ~~ "\""] :>
     Import["https://www.ncbi.nlm.nih.gov/sviewer/viewer.fcgi?id=" <>
       z <> "&db=nuccore&report=fasta", "Text"]][[1]]]
```

Data for initial images

These are from the Github site of Rallis Karamikalis.

```
In[ ]:= srules = {"Y" → "T", Except[Characters["ACGTY"]] → ""};

        ncraw[21] = Import[
            "https://github.com/rallis/intraSupplemental_Material/blob/master/code/alltogether/
              six_kingdoms/fasta/NC_000021.fasta?raw=true"];
        nc[21] = StringReplace[ncraw[21][[1]], srules];
        ncraw[913] = Import[
            "https://github.com/rallis/intraSupplemental_Material/blob/master/code/alltogether/
              six_kingdoms/fasta/NC_000913.fasta?raw=true"];
        nc[913] = StringReplace[ncraw[913][[1]], srules];
        ncraw[1136] = Import[
            "https://github.com/rallis/intraSupplemental_Material/blob/master/code/alltogether/
              six_kingdoms/fasta/NC_001136.fasta?raw=true"];
        nc[1136] = StringReplace[ncraw[1136][[1]], srules];
        ncraw[3070] = Import[
            "https://github.com/rallis/intraSupplemental_Material/blob/master/code/alltogether/
              six_kingdoms/fasta/NC_003070.fasta?raw=true"];
        nc[3070] = StringReplace[ncraw[3070][[1]], srules];
        ncraw[4317] = Import[
            "https://github.com/rallis/intraSupplemental_Material/blob/master/code/alltogether/
              six_kingdoms/fasta/NC_004317.fasta?raw=true"];
        nc[4317] = StringReplace[ncraw[4317][[1]], srules];
        ncraw[18 092] = Import[
            "https://github.com/rallis/intraSupplemental_Material/blob/master/code/alltogether/
              six_kingdoms/fasta/NC_018092.fasta?raw=true"];
        nc[18 092] = StringReplace[ncraw[18 092][[1]], srules];

In[ ]:= ncvals = {21, 913, 1136, 3070, 4317, 18 092};
        seglen = 150 000;
        chars = {"A", "T", "G", "C"};
        dim = 7;
        freq = 30;
```

Prepare FCGR images from the initial nucleotide sequences of each of the six species’ genomes.

```
In[ ]:= AbsoluteTiming[Do[
          imagesB[j] = FCGR[First[StringPartition[nc[j], seglen]], dim];
          imagesB2[j] = (# / N[Max[#]])^(1 / 5) &[imagesB[j]];
          , {j, ncvals}]]

Out[ ]= {3.063041, Null}

In[ ]:= firstImages = Map[imagesB2[#] &, ncvals];
        firstDCTs = Map[FourierDCT[#, 4][[1 ;; freq, 1 ;; freq]] &, firstImages];
```

```
images = Map[Image[#, ImageSize → 150] &, firstImages];
species = {"H. sapiens", "E. coli",
   "S. cerevisiae", "A. thalania", "P. falciparum", "P. furiosus"};
labeledImages = Thread[Labeled[images, species]];
```

In[ ]:=
```
GraphicsGrid[Partition[labeledImages, 3], ImageSize → 500]
```

In[ ]:=
```
GraphicsRow[{Style[Grid[Partition[labeledImages, 3]], FontFamily → "Times"]}]
```

Out[ ]=

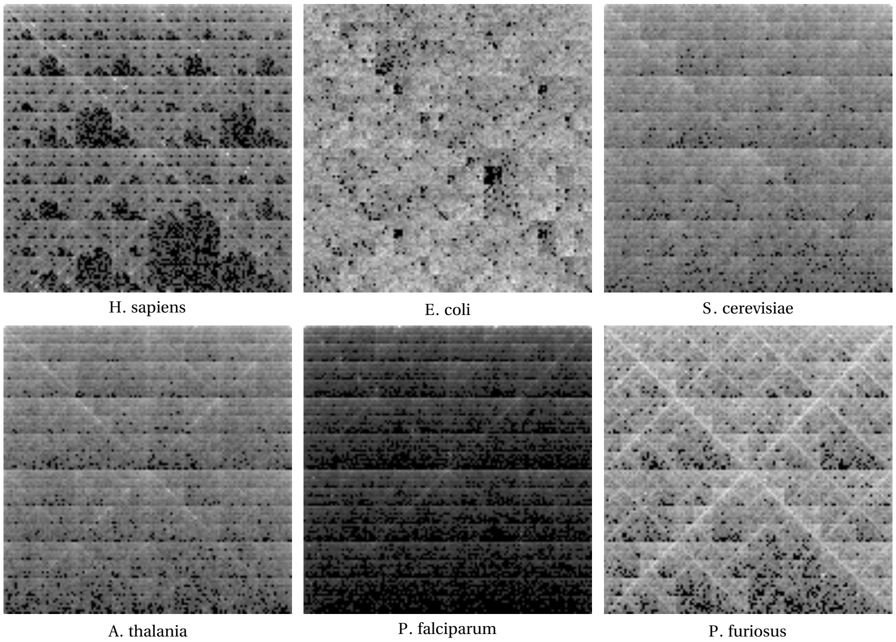

Now show the result of the Fourier Trig Transform dimension reduction (where we reverse the transform so as to view in image rather than frequency space).

In[ ]:=
```
reducedfirstImages = Map[FourierDCT[#, 4] &, firstDCTs];
reducedfirstImages2 = Map[Image[# / Max[#], ImageSize → 150] &, reducedfirstImages];
labeledReducedImages = Thread[Labeled[reducedfirstImages2, species]];
GraphicsGrid[Partition[labeledReducedImages, 3], ImageSize → 500]
```

In[ ]:=
```
GraphicsRow[{Style[Grid[Partition[labeledReducedImages,  3]], FontFamily  ->  "Times"]}]
```

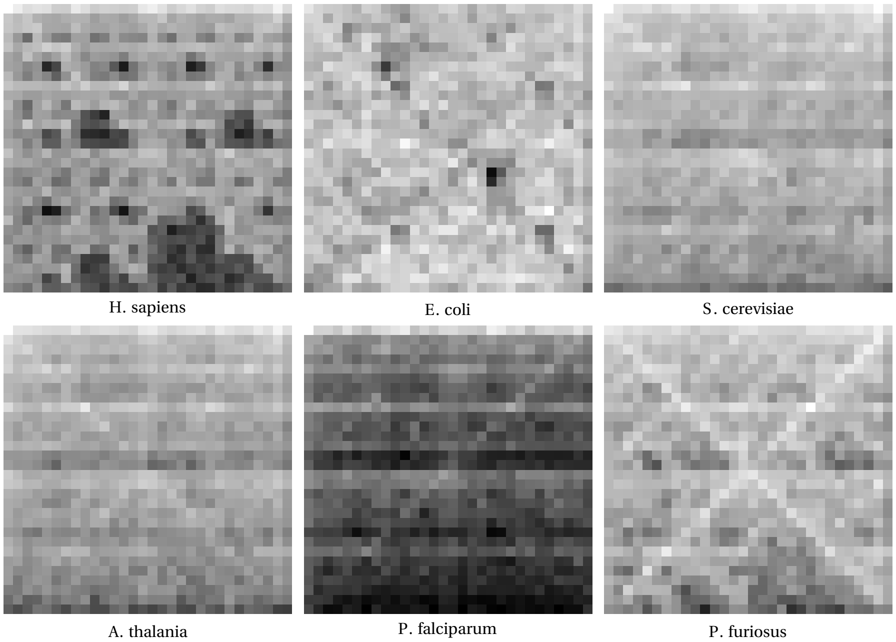

We require processing of the full set in order to see the effect of the SVD dimension reduction.

```
In[ ]:= Clear[images, images2]
AbsoluteTiming[Do[
   images[j] = Map[FCGR[#, dim] &, StringPartition[nc[j], seglen]];
   images2[j] = Map[(# / N[Max[#]])^(1 / 5) &, images[j]];
   , {j, ncvals}]]
Out[ ]= {99.840317, Null}
```

```
In[ ]:= lens = Map[Length, Table[images2[j], {j, ncvals}]]
firstPosns = Most[Prepend[Accumulate[lens], 0] + 1]
Out[ ]= {234, 30, 10, 201, 21, 12}
Out[ ]= {1, 235, 265, 275, 476, 497}
```

```
In[ ]:= allimages = Apply[Join, Map[images2, ncvals]];
speciesvals = Flatten[Table[ConstantArray[j, Length[images2[j]]], {j, ncvals}]];
AbsoluteTiming[allDCTS = Map[FourierDCT[#, 4][[1 ;; freq, 1 ;; freq]] &, allimages];]
```

```
In[ ]:= topvecs = Map[Flatten, allDCTS];
{uu, ww, vv} =
   SingularValueDecomposition[topvecs, keep];
newmat  =  uu.ww.Transpose[vv];
```

```
firstVecs = newmat[[firstPosns]];
firstImages0 = Map[Partition[#, freq] &, firstVecs];
firstImages1 = Map[FourierDCT[#, 4] &, firstImages0];
firstImages = Map[Image[# / Max[#], ImageSize → 150] &, firstImages1];
labeledReducedImages = Thread[Labeled[firstImages, species]];
```

In[ ]:=
```
GraphicsRow[{Style[Grid[Partition[labeledReducedImages, 3]], FontFamily -> "Times"]}]
```

Out[ ]=

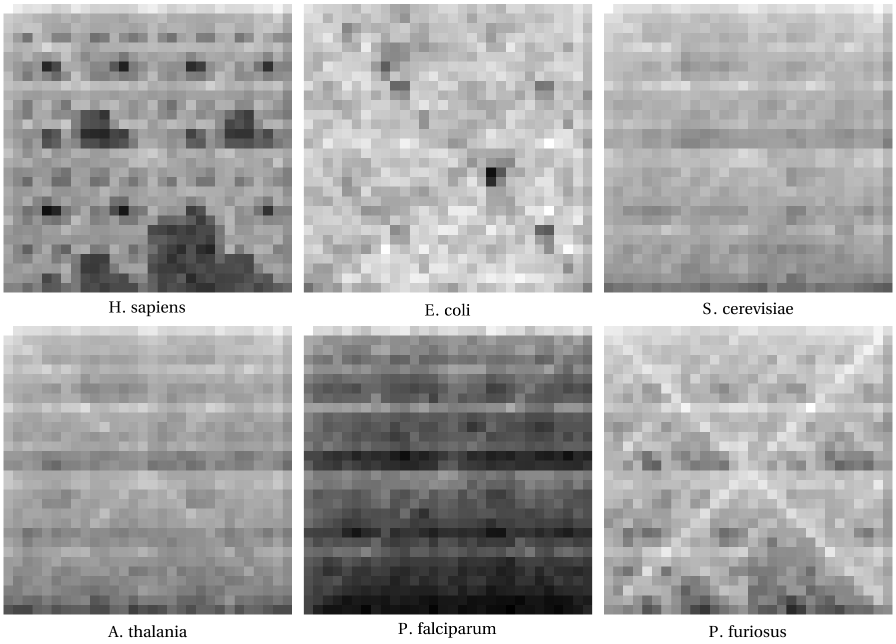

Data set from Martin Swain

The download was placed in a subdirectory ~/Notebooks/GENOMICS/gi-taxonomy-maps.clean.txt.

In[ ]:=
```
dictionaryName = FileNameJoin[
   {$HomeDirectory, "Notebooks", "GENOMICS", "gi-taxonomy-maps.clean.txt"}];
names = ReadList[dictionaryName, Record, RecordSeparators → "gi:"];
nameLists = Map[ImportString[#, "Table"] &, names][[All, 1]];
speciesAssociations = Dispatch[
   Map[#[[1]] -> SelectFirst[Rest[#], StringQ[#] && StringMatchQ[#, "species:" ~~ __] &] &,
    nameLists]];
genusAssociations = Dispatch[Map[#[[1]] -> SelectFirst[Rest[#],
       StringQ[#] && StringMatchQ[#, "genus:" ~~ __] &] &, nameLists]];
```

Read in and process the test data.

In[ ]:=
```
dim = 7; (* 6-8 *)
freq = 30;(* 20-40 *)
keep = 40;(* 24-64 *)
```

```
In[ ]:= smallFileName = FileNameJoin[
          {$HomeDirectory, "Genomes", (*"allgenomes_april_2012_v6_one_per_species.fa"*)
           "allgenomes_subset.list.fa.cleaned"}];
        j = 0;
        Clear[llRec];
        AbsoluteTiming[
          smallFTTlist = Reap[While[llRec =!= EndOfFile && MaxMemoryUsed[] ≤ 3 * 10 ^ 9,
              j ++;
              llRec = Read[smallFileName, Record, RecordSeparators → ">"];
              If[llRec === EndOfFile, Print[j]; Break[]];
              llRec = StringSplit[llRec, "\n"];
              head = llRec[[1]];
              head = StringReplace[head, {"/" → ".", ", " ~~ __ → ""}];
              head = StringReplace[head, {Whitespace → "", "|" → "_"}];
              body = Apply[StringJoin, Rest[llRec]];
              strings = StringPartition[body, seglen];
              skip = Floor[Length[strings] / 20.];
              If[skip == 0, skip = 1];
              strings = strings[[1 ;; -1 ;; skip]];
              If[Length[strings] === 0,
               Print[{j, skip, Length[strings], head, StringLength[body]}];
               Continue[]];
              speciesvals = ConstantArray[head, Length[strings]];
              Quiet[ftts = Map[processNucleotideString[#, dim, freq] &, strings]];
              PrintTemporary[
               {j, MaxMemoryUsed[], Length[strings], ByteCount[body], ByteCount[ftts]}];
              Sow[Thread[ftts -> speciesvals]];
             ]][[2, 1]];
          Close[smallFileName];
         ][[1]]

        {646, 1, 0, gi_294679205_ref_NT_167350.1_BorreliaafzeliiPKoclonefragmenta, 8579}
        {647, 1, 0, gi_294679220_ref_NT_167351.1_BorreliaafzeliiPKoclonefragmentb, 15 403}
        {648, 1, 0, gi_294679245_ref_NT_167352.1_BorreliaafzeliiPKoclonefragmentc, 6847}
        654
Out[ ]= 363.253435
```

In[ ]:=
```
smallFlatList = Flatten[smallFTTlist];
allVecs = smallFlatList[[All, 1]];
allSpecies = smallFlatList[[All, 2]];
allSpeciesIDs0 = Map[
   StringReplace[#, "gi_" ~~ dseq : DigitCharacter .. ~~ "_" ~~ __ → dseq] &, allSpecies];
allSpeciesIDs = Map[ToExpression, allSpeciesIDs0];
smallFlatListB = Transpose[{allVecs, allSpeciesIDs}];
allSpeciesList = Union[smallFlatListB[[All, 2]] /. speciesAssociations];
Length[allSpeciesList]
Union[Map[Head, allSpeciesList]]
```

Out[ ]= 129

Out[ ]= {String}

Similarly process the training data.

In[ ]:=
```
bigFileName = FileNameJoin[{$HomeDirectory, "Genomes", (*"allgenomes_april_2012_v6.fa*)
    "allgenomes_april_2012_v6_one_per_species.fa.cleaned"}];
AbsoluteTiming[
  j = 0;
  Clear[llRec];
  bigFTTlist = Reap[While[llRec =!= EndOfFile && MaxMemoryUsed[] ≤ 3 * 10 ^ 9,
      j ++;
      llRec = Read[bigFileName, Record, RecordSeparators → ">"];
      If[llRec === EndOfFile, Print[j]; Break[]];
      llRec = StringSplit[llRec, "\n"];
      head = llRec[[1]];
      head = StringReplace[head, {"/" → ".", ", " ~~ __ → ""}];
      head = StringReplace[head, {Whitespace → "", "|" → "_"}];
      body = Apply[StringJoin, Rest[llRec]];
      strings = StringPartition[body, seglen];
      skip = Floor[Length[strings] / 20.];
      If[skip == 0, skip = 1];
      strings = strings[[1 ;; -1 ;; skip]];
      If[Length[strings] === 0, Continue[]];
      speciesvals = ConstantArray[head, Length[strings]];
      Quiet[ftts = Map[processNucleotideString[#, dim, freq] &, strings]];
      PrintTemporary[
       {j, MaxMemoryUsed[], Length[strings], ByteCount[body], ByteCount[ftts]}];
      Sow[Thread[ftts -> speciesvals]];
     ]][[2, 1]];
  Close[bigFileName];
 ][[1]]
```

```
1053
```

Out[ ]= 584.953518

In[ ]:=
```
fullFlatList = Flatten[bigFTTlist];
fullVecs = fullFlatList[[All, 1]];
fullSpecies = fullFlatList[[All, 2]];
fullSpeciesIDs0 = Map[
   StringReplace[#, "gi_" ~~ dseq : DigitCharacter .. ~~ "_" ~~ __ → dseq] &, fullSpecies];
fullSpeciesIDs = Map[ToExpression, fullSpeciesIDs0];
fullFlatListB = Transpose[{fullVecs, fullSpeciesIDs}];
fullSpeciesList = Union[fullFlatListB[[All, 2]] /. speciesAssociations];
Length[fullSpeciesList]
Union[Map[Head, fullSpeciesList]]
```

Out[ ]= 565

Out[ ]= {String}

Remove species instances in test list that do not appear in full list

In[ ]:=
```
Clear[inList];
Scan[(inList[#] = True) &, fullSpeciesList];
{allVecsReduced, allSpeciesIDsReduced} =
  Transpose[Reap[Do[If[inList[smallFlatListB[[j, 2]] /. speciesAssociations],
        Sow[{allVecs[[j]], allSpeciesIDs[[j]]}];
       ];
      , {j, Length[allVecs]}]][[2, 1]]];
```

Now do the SVD step on the training data, create a kd tree, and use the right multiplier matrix from the SVD to preprocess the test data.

In[ ]:=
```
keep = 40;
Timing[{nf, vv} = imageKNN[fullVecs, fullSpeciesIDs, keep];]
Timing[testVecs = processTestInput[allVecsReduced, vv];]
MaxMemoryUsed[]
```

Out[ ]= {6.48, Null}

Out[ ]= {0.088, Null}

Out[ ]= 1 005 601 408

Perform the lookup.

```
In[ ]:= nbrCount = 20;
Timing[nearSegments = Map[nf[#, nbrCount] &, testVecs];]
nbrListG = Transpose[{allSpeciesIDsReduced, nearSegments}] /. genusAssociations;
directhitsG = Cases[nbrListG, {aa_, {aa_, ___}}];
hitsG = Cases[nbrListG, {aa_, {___, aa_, ___}}];
{Length[directhitsG], Length[hitsG], Length[nbrListG],
 N[Length[directhitsG] / Length[nbrListG]], N[Length[hitsG] / Length[nbrListG]]}
nbrListS = Transpose[{allSpeciesIDsReduced, nearSegments}] /. speciesAssociations;
directhitsS = Cases[nbrListS, {aa_, {aa_, ___}}];
hitsS = Cases[nbrListS, {aa_, {___, aa_, ___}}];
{Length[directhitsS], Length[hitsS], Length[nbrListS],
 N[Length[directhitsS] / Length[nbrListS]], N[Length[hitsS] / Length[nbrListS]]}

Out[ ]= {2.848, Null}

Out[ ]= {13 119, 13 965, 14 339, 0.914917358254, 0.973917288514}

Out[ ]= {11 893, 13 693, 14 339, 0.829416277286, 0.954948043797}
```

Now aggregate by genome rather than gene fragment. Use majority to determine the best guesses for genus and species.

```
Clear[inList];
Scan[(inList[#] = True) &, fullSpeciesList];
allGenes = Reap[Do[If[inList[smallFlatListB[[j, 2]] /. speciesAssociations],
        Sow[{processTestInput[{allVecs[[j]]}, vv], allSpeciesIDs[[j]]}];
       ];
      , {j, Length[allVecs]}]][[2, 1]];
groupedGenes = SplitBy[allGenes, #[[2]] &];
```

```
In[ ]:= Timing[nearSegmentsByGene =
    Map[Flatten, Map[First[nf[#, 1]] &, groupedGenes[[All, All, 1]], {2}]];]
nearSegmentsByGeneB = Map[First[Commonest[#]] &, nearSegmentsByGene];
nbrListG =
  Transpose[{groupedGenes[[All, 1, 2]], nearSegmentsByGeneB}] /. genusAssociations;
directhitsG = Cases[nbrListG, {aa_, aa_}];
{Length[directhitsG], Length[nbrListG], N[Length[directhitsG] / Length[nbrListG]]}
nbrListS =
  Transpose[{groupedGenes[[All, 1, 2]], nearSegmentsByGeneB}] /. speciesAssociations;
directhitsS = Cases[nbrListS, {aa_, aa_}];
{Length[directhitsS], Length[nbrListS], N[Length[directhitsS] / Length[nbrListS]]}

Out[ ]= {1.388, Null}

Out[ ]= {617, 640, 0.9640625}

Out[ ]= {576, 640, 0.9}
```

Use a neural net.

```
keep = 80;
Timing[{trainData, vv} = trainImageProcess[fullVecs, keep];]
trainData = trainData -> fullSpeciesIDs;
Timing[testVecs = testImageProcess[allVecsReduced, vv];]
speciesIDs = Union[fullSpeciesIDs];
labelLen = Length[speciesIDs];
dictionary = Thread[speciesIDs → Range[labelLen]];
reverseDictionary = Thread[Range[labelLen] -> speciesIDs];
```

```
In[ ]:= net = NetChain[{400, Ramp, Tanh, labelLen, Tanh, SoftmaxLayer[]},
    "Input" → (keep + 1), "Output" → NetDecoder[{"Class", Range[labelLen]}]];
AbsoluteTiming[trained = NetTrain[net, trainData /. dictionary, MaxTrainingRounds → 200,
     LossFunction -> CrossEntropyLossLayer["Index"], Method → {"ADAM", "Beta1" → .9}];]

Out[ ]= {1141.201573, Null}
```

```
In[ ]:= AbsoluteTiming[resNN = Map[trained, testVecs];]
genusPairs =
  Transpose[{allSpeciesIDsReduced, resNN}] /. reverseDictionary /. genusAssociations;
speciesPairs = Transpose[{allSpeciesIDsReduced, resNN}] /. reverseDictionary /.
    speciesAssociations;
{Length[allSpeciesIDsReduced], Length[Cases[genusPairs, {a_, a_}]],
 Length[Cases[speciesPairs, {a_, a_}]],
 N[Length[Cases[genusPairs, {a_, a_}]] / Length[allSpeciesIDsReduced]],
 N[Length[Cases[speciesPairs, {a_, a_}]] / Length[allSpeciesIDsReduced]]}

Out[ ]= {2.652325, Null}

Out[ ]= {14 339, 13 407, 12 234, 0.935002440895, 0.853197573053}
```

Randhawa, Hill, Kari Cyprinidae data set

```
In[ ]:= url =
  "https://raw.githubusercontent.com/grandhawa/MLDSP/master/DataBase/FamilyToGenus(
    Cyprinidae)";
filesAcheilognathus = Table["Acheilognathus/C" ~~ ToString[j] ~~ ".txt", {j, 1, 10}];
filesAcrossocheilus = Table["Acrossocheilus/C" ~~ ToString[j] ~~ ".txt", {j, 60, 71}];
filesLabeo = Table["Labeo/C" ~~ ToString[j] ~~ ".txt", {j, 41, 59}];
filesOnychostoma = Table["Onychostoma/C" ~~ ToString[j] ~~ ".txt", {j, 72, 81}];
filesRhodeus = Table["Rhodeus/C" ~~ ToString[j] ~~ ".txt", {j, 11, 21}];
filesSchizothorax = Table["Schizothorax/C" ~~ ToString[j] ~~ ".txt", {j, 22, 40}];
```

```
genusNames = {"Acheilognathus", "Acrossocheilus",
   "Labeo", "Onychostoma", "Rhodeus", "Schizothorax"};
sequencesAcheilognathus = Map[StringJoin[Import[URL[URLBuild[{url, #}]], "FASTA"]] &,
   filesAcheilognathus];
sequencesAcrossocheilus = Map[Import[URLBuild[{url, #}], "Text"] &, filesAcrossocheilus];
sequencesLabeo = Map[Import[URLBuild[{url, #}], "Text"] &, filesLabeo];
sequencesOnychostoma = Map[Import[URLBuild[{url, #}], "Text"] &, filesOnychostoma];
sequencesRhodeus = Map[Import[URLBuild[{url, #}], "Text"] &, filesRhodeus];
sequencesSchizothorax = Map[Import[URLBuild[{url, #}], "Text"] &, filesSchizothorax];
sequences = {sequencesAcheilognathus, sequencesAcrossocheilus, sequencesLabeo,
   sequencesOnychostoma, sequencesRhodeus, sequencesSchizothorax};
```

```
In[ ]:= Map[Length, sequences]
MinMax[Map[StringLength, sequences, {2}]]
Out[ ]= {10, 12, 19, 10, 11, 19}
Out[ ]= {16 563, 17 155}
```

```
In[ ]:= dim = 7;
freq = 30;
ftts =
  Map[Developer`ToPackedArray[processNucleotideString[#, dim, freq]] &, sequences, {2}];
SeedRandom[1 144 477];
trainFraction = 75 / 100;
keep = 36;
nbrCount = 2;
```

Run 1000 randomized trials with 75% in each genus for

```
results = Table[
   trainFTTIndices =
    Map[Sort[RandomSample[Range[Length[#]], Floor[trainFraction * Length[#]]]] &, ftts];
   testFTTIndices = Table[Complement[Range[Length[ftts[[j]]]], trainFTTIndices[[j]]],
     {j, Length[trainFTTIndices]}];
   trainFTTs = Flatten[Table[ftts[[j, trainFTTIndices[[j]]]], {j, Length[ftts]}], 1];
   testFTTs = Flatten[Table[ftts[[j, testFTTIndices[[j]]]], {j, Length[ftts]}], 1];
   trainGenera = Flatten[Table[ConstantArray[genusNames[[j]], Length[trainFTTIndices[[j]]]],
      {j, Length[trainFTTIndices]}]];
   testGenera = Flatten[Table[ConstantArray[genusNames[[j]], Length[testFTTIndices[[j]]]],
      {j, Length[testFTTIndices]}]];
   {nf, vv} = imageKNN[trainFTTs, trainGenera, keep];
   testVecs = processTestInput[testFTTs, vv];
   nearSegments = Map[nf[#, nbrCount] &, testVecs];
   nbrListG = Transpose[{testGenera, nearSegments}];
   directhitsG = Cases[nbrListG, {aa_, {aa_, ___}}];
   hitsG = Cases[nbrListG, {aa_, {___, aa_, ___}}];
   {Length[directhitsG], Length[hitsG], Length[nbrListG],
    N[Length[directhitsG] / Length[nbrListG]], N[Length[hitsG] / Length[nbrListG]]},
   1000];
Total[results]
```

Out[ ]= {21 145, 21 700, 22 000, 961.136363636, 986.363636364}

Phylogenetic trees

Influenza A

```
In[ ]:= speciesInfluenzaA = {"A/turkey/Ontario/FAV110-4/2009(H1N1)",
   "A/mallard/Nova Scotia/00088/2010(H1N1)",
   "A/thick-billed murre/Canada/1871/2011(H1N1)",
   "A/duck/Guangxi/030D/2009(H1N1)",
   "A/mallard/France/691/2002(H1N1)",
   "A/duck/Hokkaido/w73/2007(H1N1)",
   "A/pintail/Miyagi/1472/2008(H1N1)",
   "A/mallard/Korea/KNU YP09/2009(H1N1)",
   "A/mallard/Maryland/352/2002(H1N1)",
   "A/mallard/Maryland/26/2003(H1N1)",
   "A/dunlin/Alaska/44421-660/2008(H1N1)",
   "A/mallard/Minnesota/Sg-00620/2008(H1N1)",
   "A/turkey/Virginia/4135/2014(H1N1)",
   "A/chicken/Eastern China/XH222/2008(H5N1)",
   "A/duck/Eastern China/JS017/2009(H5N1)",
   "A/chicken/Yunnan/chuxiong01/2005(H5N1)",
   "A/chicken/Germany/R3234/2007(H5N1)",
   "A/domestic duck/Germany/R1772/2007(H5N1)",
   "A/wild bird/Hong Kong/07035-1/2011(H5N1)",
   "A/Chicken/Hong Kong/822.1/01 (H5N1)",
   "A/chicken/Miyazaki/10/2011(H5N1)",
   "A/chicken/Korea/es/2003(H5N1)",
   "A/mandarin duck/Korea/K10-483/2010(H5N1)",
   "A/turkey/VA/505477-18/2007(H5N1)",
   "A/American black duck/NB/2538/2007(H7N3)",
   "A/American black duck/New Brunswick/02490/2007(H7N3)",
   "A/American green-winged teal/California/44242-906/2007(H7N3)",
   "A/avian/Delaware Bay/226/2006(H7N3)",
   "A/chicken/British Columbia/GSC_human_B/04(H7N3)",
   "A/chicken/Rizhao/713/2013(H7N9)",
   "A/chicken/Jiangsu/1021/2013(H7N9)",
   "A/duck/Jiangxi/3096/2009(H7N9)",
   "A/wild duck/Korea/SH19-47/2010(H7N9)",
   "A/turkey/Minnesota/1/1988(H7N9)",
   "A/mallard/Minnesota/AI09-3770/2009(H7N9)",
   "A/mallard/Postdam/178-4/1983(H2N2)",
   "A/duck/Hong Kong/319/1978(H2N2)",
   "A/emperor goose/Alaska/44297-260/2007(H2N2)"
   };
```

```
In[ ]:= genomesInfluenzaA = {"HM370969", "CY138562", "CY149630", "KC608160", "AM157358",
    "AB470663", "AB546159", "HQ897966", "EU026046", "FJ357114", "GQ411894", "CY140047",
    "KM244078", "HQ185381", "HQ185383", "EU635875", "FM177121", "AM914017", "KF572435",
    "AF509102", "AB684161", "EF541464", "JF699677", "GU186511", "EU500854", "CY129336",
    "CY076231", "CY039321", "AY646080", "KF259734", "KF938945", "KF259688",
    "KC609801", "CY014788", "CY186004", "DQ017487", "CY005540", "JX081142"};

In[ ]:= AbsoluteTiming[sequencesInfluenzaA = Map[getfasta, genomesInfluenzaA];]

Out[ ]= {54.314014, Null}

In[ ]:= stringColor[str_String] := Which[
   StringContainsQ[str, "H1N1"], Darker[Red],
   StringContainsQ[str, "H5N1"], Blue,
   StringContainsQ[str, "H7N3"], Pink,
   StringContainsQ[str, "H7N9"], Darker[Green],
   StringContainsQ[str, "H2N2"], Black]
speciesInfluenzeColored = Map[Style[#, stringColor[#]] &, speciesInfluenzaA];

In[ ]:= dim = 7;
freq = 30;
keep = 40;
fttsInfluenzaA = Map[Developer`ToPackedArray[processNucleotideString[#, dim, freq]] &,
    sequencesInfluenzaA];
vecsInfluenzaA = trainImageProcessB[fttsInfluenzaA, keep];

In[ ]:= DendrogramPlot[vecsInfluenzaA,
  LeafLabels -> speciesInfluenzeColored, Orientation -> Left,
  AspectRatio -> 4.5, ImageSize → 500, DistanceFunction → CosineDistance]
```

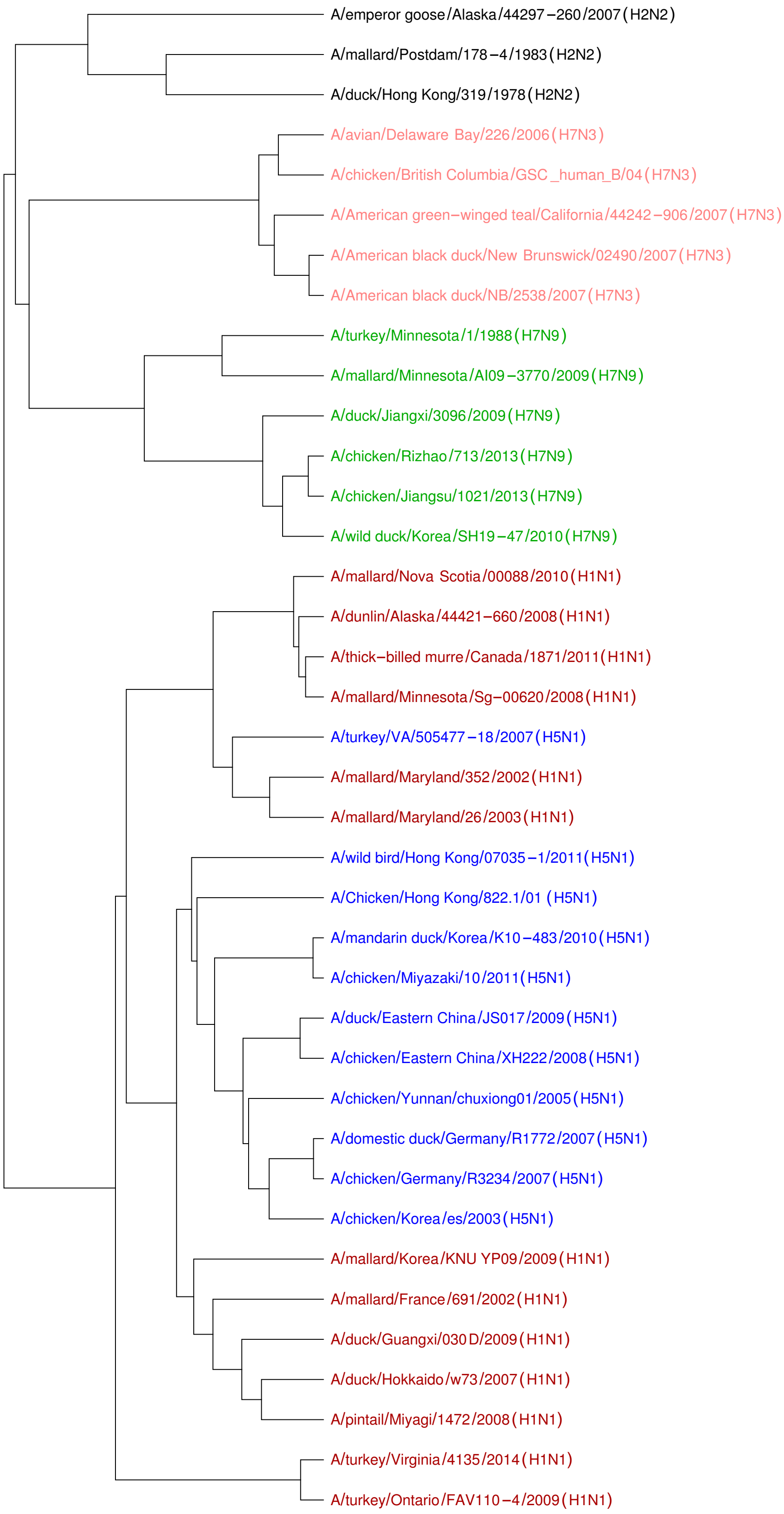

Compute MDS for plotting

```
diffs = Table[vecj - veck, {vecj, vecsInfluenzaA}, {veck, vecsInfluenzaA}];
dist2mat = -Map[#.# &, diffs, {2}]/ 2;
len = Length[vecsInfluenzaA];
onevec = ConstantArray[{1}, len];
hmat = IdentityMatrix[len] - onevec.Transpose[onevec]/ len;
bmat = hmat.dist2mat.hmat;
{uu, ww, vv} = SingularValueDecomposition[bmat, 3];
newvals3 = uu. Sqrt[ww];
```

MDS plots

```
ListPointPlot3D[newvals3 -> speciesInfluenzeColored, ImageSize → 500,
 LabelingFunction → Callout, LabelingSize → Large, PlotLabel -> "Influenza A MDS plot"]
```

Influenza A MDS plot

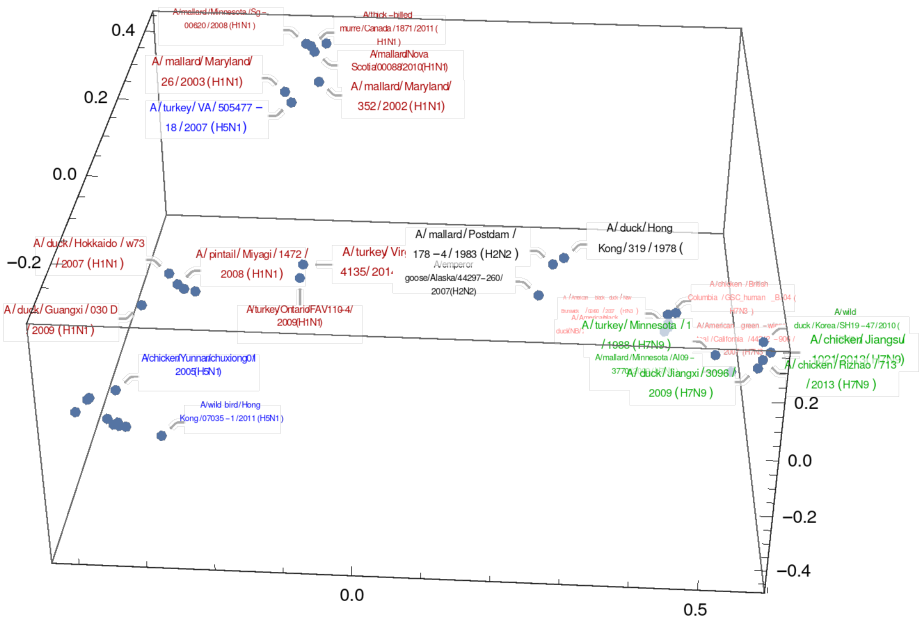

In[ ]:= `ListPointPlot3D[colorPointList, ImageSize → 320]`

Out[ ]=
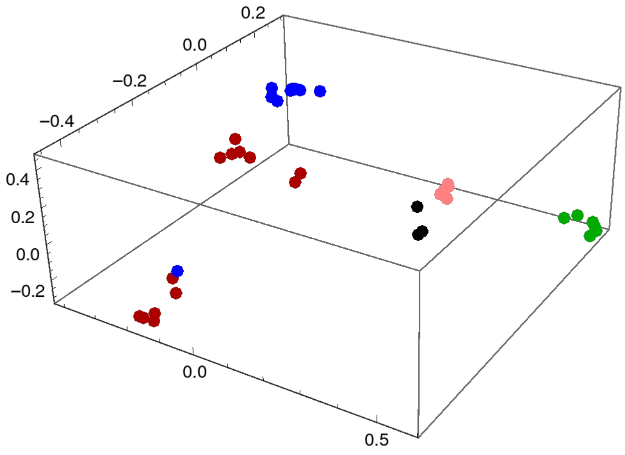

Simple GCGR images of four H1N1 sequences and 4 H5N1 sequences, with the “misplaced” one last.

```
dim = 5;
keep = {4, 6, 9, 10, 21, 22, 23, 24};
fcgrArrays = Map[FCGR[#, dim] &, sequencesInfluenzaA[[keep]]];
fcgrArrays = Map[(N[#] / Max[#]) ^ (1 / 5) &, fcgrArrays];
imagesViral = Map[Image[#, ImageSize → 100] &, fcgrArrays];
labeledViralImages = Thread[Labeled[imagesViral,
    Join[ConstantArray["H1N1", 4], ConstantArray["H5N1", 3], {"misplaced H5N1"}]]];

GraphicsRow[{Style[Grid[Partition[labeledViralImages, 4]], FontFamily -> "Times"]}]
```

Out[ ]=

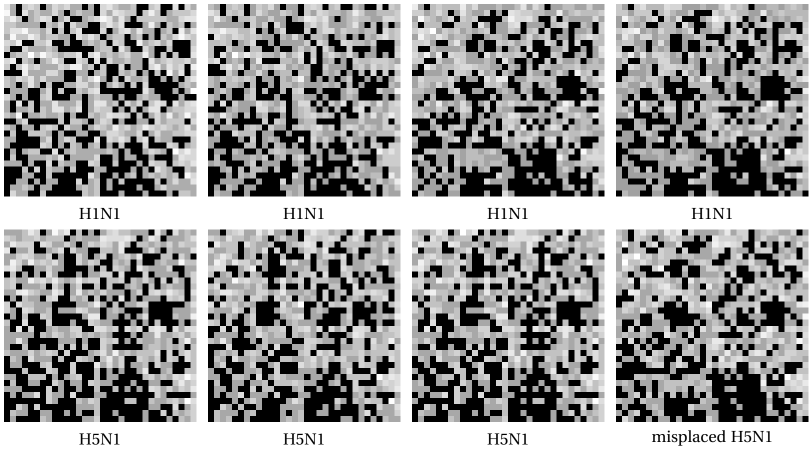

Take image differences from each with the last one:

```
In[ ]:= imagesViralDiffs = Map[ImageDifference[#, Last[imagesViral]] &, imagesViral];
labeledImageDiffs = Thread[Labeled[imagesViralDiffs,
    Join[ConstantArray["H1N1", 4], ConstantArray["H5N1", 3], {"misplaced H5N1"}]]];

In[ ]:= GraphicsRow[{Style[Grid[Partition[labeledImageDiffs, 4]], FontFamily -> "Times"]}]
```

Out[ ]=

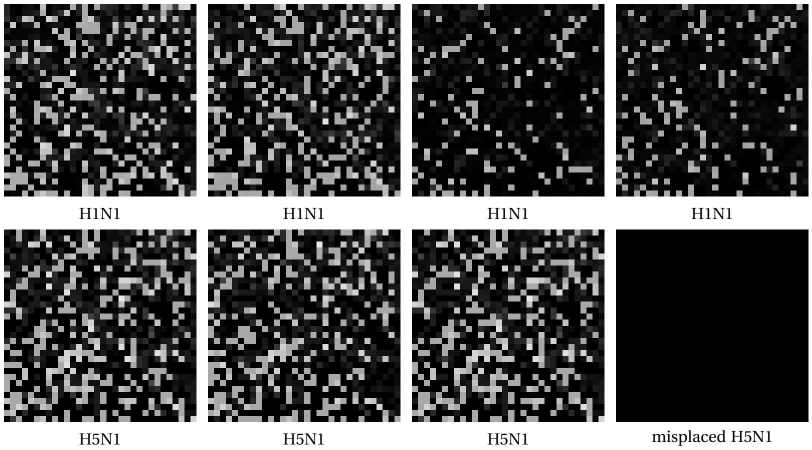

Eukaryotic mitochondrial DNA

In[ ]:=
```
speciesMitochondrial = {"protozoa", "fungus", "alga", "yeastA", "yeastB",
   "plant", "roundworm", "shrimp", "urchinA", "urchinB", "fruitfly",
   "honeybee", "mosquito", "chicken", "carp", "loach", "trout", "opossum",
   "whaleA", "whaleB", "sealA", "sealB", "cow", "rat", "mouse", "human"};
genomesMitochondrial = {"X15917", "M61734", "U02970", "X54421", "M62622",
   "M68929", "X54253", "X69067", "J04815", "X12631", "X03240", "L06178",
   "L20934", "X52392", "X61010", "M91245", "L29771", "Z29573", "X61145",
   "X72204", "X72004", "X63726", "J01394", "X14848", "V00711", "J01415"};
```

In[ ]:=
```
speciesColors = {Lighter[Blue], Lighter[Blue], Lighter[Blue], Lighter[Blue],
   Lighter[Blue], Blue, Orange, Darker[Red], Lighter[Red], Lighter[Red],
   Green, Green, Green, Darker[Brown], Darker[Orange], Darker[Orange],
   Darker[Orange], Darker[Brown], Lighter[Black], Lighter[Black], Lighter[Black],
   Lighter[Black], Lighter[Black], Darker[Brown], Darker[Brown], Black};
speciesMitochondrialColored = Table[Style[speciesMitochondrial[[j]], speciesColors[[j]]],
   {j, Length[speciesMitochondrial]}];
```

In[ ]:=
```
AbsoluteTiming[sequencesMitochondrial = Map[getfasta, genomesMitochondrial];]
```

Out[ ]=
```
{36.858867, Null}
```

In[ ]:=
```
Map[StringLength, sequencesMitochondrial]
```

Out[ ]=
```
{41107, 101829, 56186, 19775, 79757, 189349, 14542, 16106,
 15984, 15944, 16308, 16642, 15641, 17060, 16868, 16856, 16940,
 17390, 16695, 16703, 17096, 17127, 16624, 16582, 16572, 16853}
```

In[ ]:=
```
Map[Dimensions, vecsMitochondrial]
```

Out[ ]=
```
{{26, 27}, {900, 26}}
```

vecsMitochondrial

```
In[ ]:= AbsoluteTiming[dim = 7;
  freq = 30;
  keep = 40;
  fttsMitochondrial =
   Map[Developer`ToPackedArray[processNucleotideString[#, dim, freq]] &,
    sequencesMitochondrial];
  vecsMitochondrial = trainImageProcessB[fttsMitochondrial, keep];
  dd = DendrogramPlot[vecsMitochondrial,
    DistanceFunction → CosineDistance, LeafLabels → speciesMitochondrialColored,
    Orientation → Left, AspectRatio → 1.2, ImageSize → 400];]
dd

Out[ ]= {1.176484, Null}

Out[ ]=
```

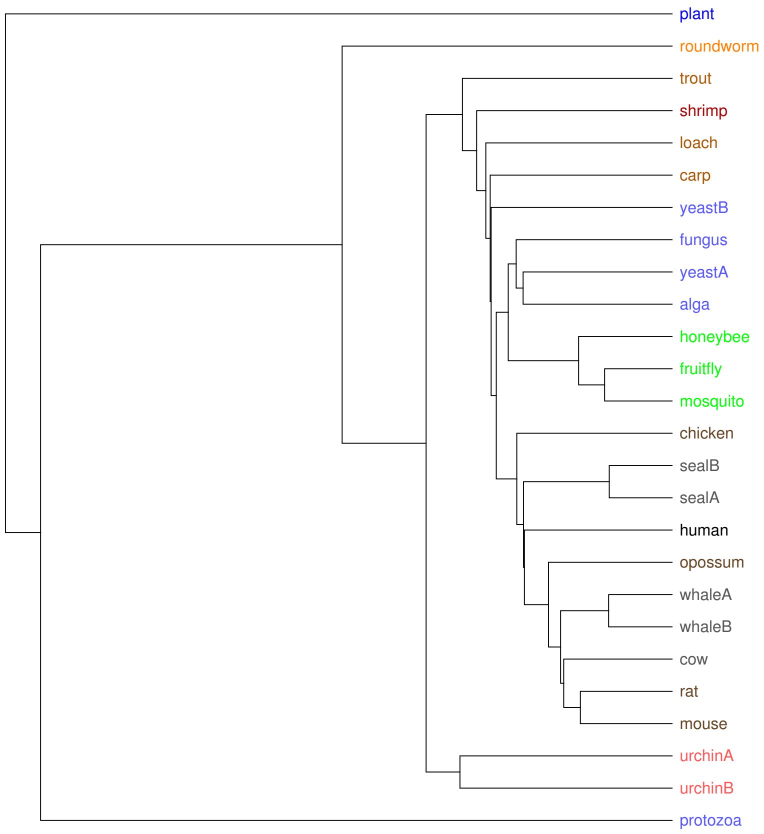

Here we make a multidimensional scaling (MDS) plot.

In[ ]:=
```
diffs = Table[vecj - veck, {vecj, vecsMitochondrial}, {veck, vecsMitochondrial}];
dist2mat = -Map[#.# &, diffs, {2}] / 2;
len = Length[vecsMitochondrial];
onevec = ConstantArray[{1}, len];
hmat = IdentityMatrix[len] - onevec.Transpose[onevec] / len;
bmat = hmat.dist2mat.hmat;
{uu, ww, vv} = SingularValueDecomposition[bmat, 2];
newvals2 = uu. Sqrt[ww];
```

In[ ]:=
```
ListPlot[newvals2 -> speciesMitochondrialColored,
 LabelingFunction → Callout, ImageSize → 400]
```

Out[ ]=

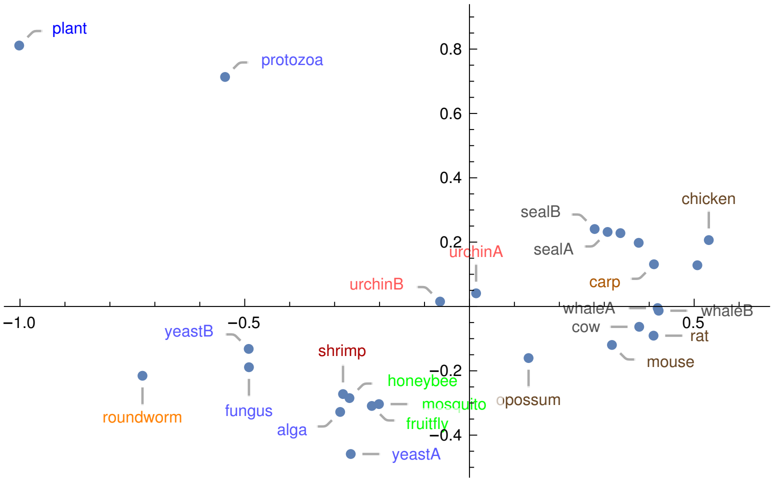

Mammalian mitochondrial DNA

In[ ]:=
```
genomesMammal = {"V00662", "D38116", "D38113", "D38114", "X99256", "Y18001",
  "AY863426", "D38115", "NC_002083", "U20753", "U96639", "AJ002189",
  "AF010406", "AF533441", "V00654", "AY488491", "EU442884", "EF551003",
  "EF551002", "X97336", "Y07726", "DQ402478", "AF303110", "AF303111",
  "EF212882", "AJ001588", "X88898", "NC_002764", "AJ238588", "AJ001562",
  "X72204", "NC_005268", "NC_007441", "NC_008830", "NC_001788", "NC_001321",
  "NC_005270", "NC_001640", "NC_005275", "NC_006931", "NC_010640"};
```

In[ ]:=
```
speciesMammal = {"Human", "Pigmy chimpanzee", "Common chimpanzee",
   "Gorilla", "Gibbon", "Baboon", "Vervet monkey", "Bornean orangutan",
   "Sumatran orangutan", "Cat", "Dog", "Pig", "Sheep", "Goat", "Cow", "Buffalo",
   "Wolf", "Tiger", "Leopard", "Indian rhinoceros", "White rhinoceros",
   "Black bear", "Brown bear", "Polar bear", "Giant panda", "Rabbit", "Hedgehog",
   "Macaca thibet", "Squirrel", "Dormouse", "Blue whale", "Bowhead whale",
   "Chiru", "Common warthog", "Donkey", "Fin whale", "Gray whale", "Horse",
   "Indus river dolphin", "North pacific right whale", "Taiwan serow"};
```

In[ ]:=
```
colorsMammal = Join[ConstantArray[Red, 4], ConstantArray[Lighter[Red], 3],
    {Red, Red}, {Darker[Blue, .35], Blue}, ConstantArray[Lighter[Brown], 5],
    {Blue, Darker[Blue, .35], Darker[Blue, .35]}, {Darker[Brown, .4], Darker[Brown, .4]},
    ConstantArray[Darker[Blue, .7], 4], {Lighter[Black, .2], Lighter[Black, .4]},
    {Lighter[Red]}, {Black, Black}, {Darker[Green], Darker[Green]},
    {Lighter[Brown], Lighter[Brown]}, {Darker[Brown, .4]}, {Darker[Green], Darker[Green]},
    {Darker[Brown, .4]}, {Darker[Green], Darker[Green]}, {Lighter[Brown]}];
speciesMammalColored = Table[Style[speciesMammal[[j]], colorsMammal[[j]]],
    {j, Length[speciesMammal]}];
```

In[ ]:=
```
AbsoluteTiming[sequencesMammal = Map[getfasta, genomesMammal];]
```

Out[ ]=
```
{59.400678, Null}
```

In[ ]:=
```
AbsoluteTiming[
  dim = 7;
  freq = 30;
  keep = 40;
  fttsMammal =
   Map[Developer`ToPackedArray[processNucleotideString[#, dim, freq]] &, sequencesMammal];
  vecsMammal = trainImageProcessB[fttsMammal, keep];
  dd = DendrogramPlot[vecsMammal, LeafLabels → speciesMammalColored, DistanceFunction →
     CosineDistance, Orientation → Left, AspectRatio → 1.2, ImageSize → 450];]
dd
```

Out[ ]= {1.139126, Null}

Out[ ]=

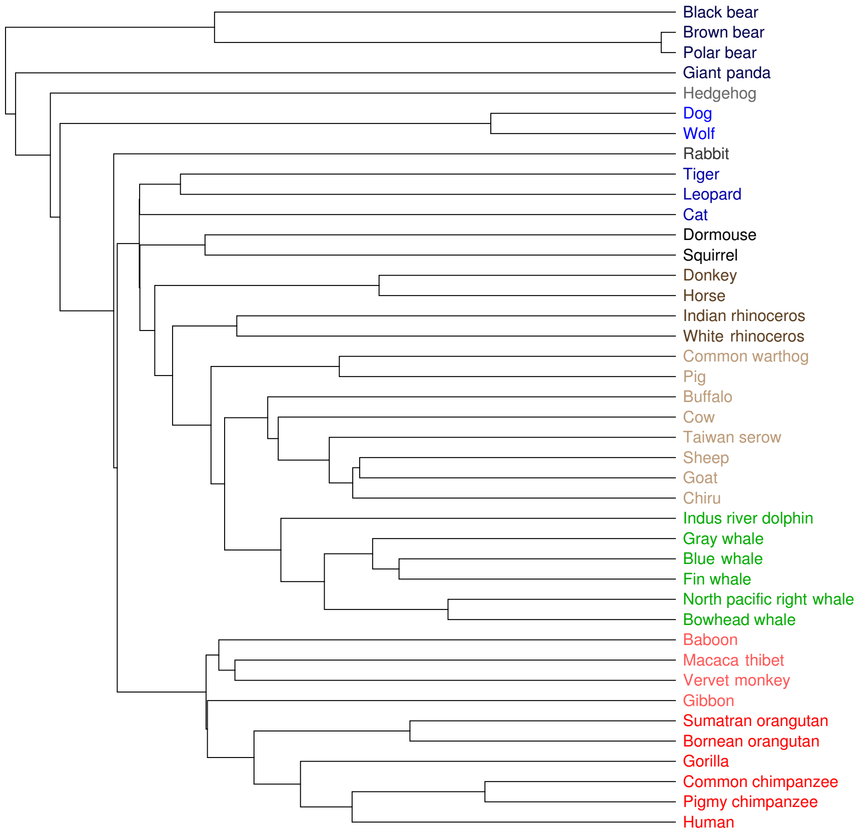

2D and 3D MDS plots

In[ ]:=
```
diffs = Table[vecj - veck, {vecj, vecsMammal}, {veck, vecsMammal}];
dist2mat = -Map[#.# &, diffs, {2}] / 2;
len = Length[vecsMammal];
onevec = ConstantArray[{1}, len];
hmat = IdentityMatrix[len] - onevec.Transpose[onevec] / len;
bmat = hmat.dist2mat.hmat;
{uu, ww, vv} = SingularValueDecomposition[bmat, 2];
newvals2 = uu. Sqrt[ww];
```

In[ ]:=
```
ListPlot[newvals2 -> speciesMammalColored, LabelingFunction → Callout, ImageSize → 400]
```

Out[ ]=

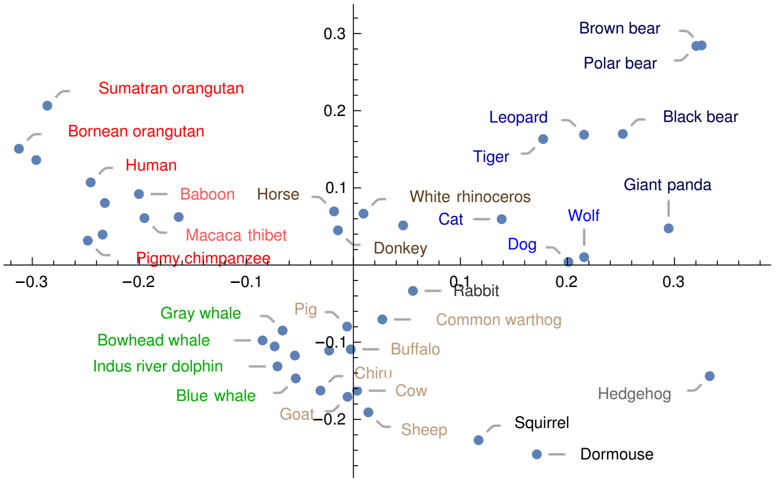

In[ ]:=
```
diffs = Table[vecj - veck, {vecj, vecsMammal}, {veck, vecsMammal}];
dist2mat = -Map[#.# &, diffs, {2}] / 2;
len = Length[vecsMammal];
onevec = ConstantArray[{1}, len];
hmat = IdentityMatrix[len] - onevec.Transpose[onevec] / len;
bmat = hmat.dist2mat.hmat;
{uu, ww, vv} = SingularValueDecomposition[bmat, 3];
newvals3 = uu. Sqrt[ww];
```

In[ ]:=
```
ListPointPlot3D[newvals3 -> speciesMammalColored,
  LabelingFunction → Callout, ImageSize → 400]
```

Out[ ]=

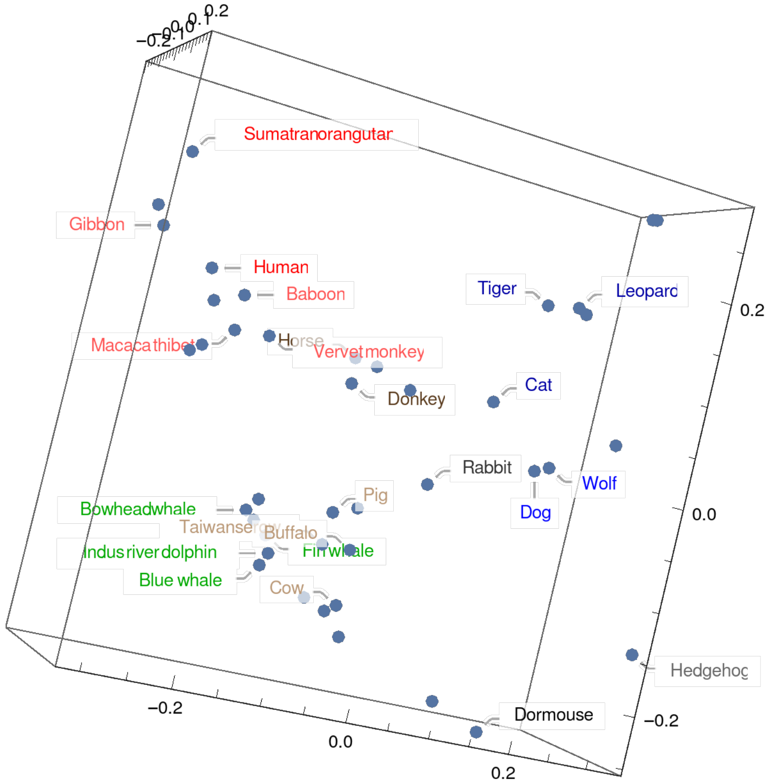

Bacterial hosts of viral phages

Import the data files and split off the 820 viruses from the 2699 candidate bacteria hosts.

```
dim = 7;
freq = 30;
phageLength = 820;
allGenomes = Import[
   "https://raw.githubusercontent.com/linsalrob/PhageHosts/master/data/all_host_taxid
     _taxonomy.txt", "Data"];
{phageGenomes, bacteriaGenomes} = TakeDrop[allGenomes, phageLength];
phageIDs = phageGenomes[[All, 1]];
Union[Map[StringMatchQ[#, "NC_*"] &, phageIDs]]
bacteriaIDs = bacteriaGenomes[[All, 1]];
bacteriaLength = Length[bacteriaIDs]
Union[Map[StringMatchQ[#, "NC_*"] &, bacteriaIDs]]
Position[Map[StringMatchQ[#, "NC_*"] &, bacteriaIDs], False]
bacteriaIDs[[FirstPosition[Map[StringMatchQ[#, "NC_*"] &, bacteriaIDs], False]]]
phageHostGenera0 = Map[StringSplit[#[[3]], ";"][[-2]] &, phageGenomes];
bacteriaGenera0 = Map[StringSplit[#[[3]], ";"][[-2]] &, bacteriaGenomes];
phageHostGenera = Map[If[# === " ", "Unknown", StringSplit[#][[1]]] &, phageHostGenera0];
bacteriaGenera = Map[If[# === " ", "Unknown", StringSplit[#][[1]]] &, bacteriaGenera0];
```

Process the viral sequences.

```
In[ ]:= AbsoluteTiming[phageFTTs = Table[
     phageDNA = getfasta[phageIDs[[j]]];
     If[Mod[j, 10] == 0, PrintTemporary[{j, StringLength[phageDNA]}]];
     processNucleotideString[phageDNA, dim, freq]
     , {j, Length[phageIDs]}];]

Out[ ]= {948.888923, Null}
```

Split the bacterial sequences into chunks of length 20000 and process those. For the (small) subset that have sequences smaller than that size, use the entire sequence.

```
seqLength = 20 000;(*20000*)
AbsoluteTiming[bacteriaFTTs = Table[
    bacteriaDNA = StringReplace[getfasta[bacteriaIDs[[j]]], srules];
    bacteriaDNASequences =
     If[! StringQ[bacteriaDNA], {""}, If[StringLength[bacteriaDNA] <= seqLength,
       {bacteriaDNA}
       ,
       StringPartition[bacteriaDNA, seqLength]
      ]];
    If[Mod[j, 10] == 0,
     PrintTemporary[{j, StringLength[bacteriaDNA], Length[bacteriaDNASequences]}]];
    ftts = If[bacteriaDNASequences === {""},
      {},
      Map[processNucleotideString[#, dim, freq] &, bacteriaDNASequences]
     ]
    , {j, 1, bacteriaLength}];]
```

Out[ ]= {17 525.699385, Null}

Put together the bacterial vectors and corresponding genus names.

```
generaLists = Table[
   ConstantArray[bacteriaGenera[[j]], Length[bacteriaFTTs[[j]]]], {j, Length[bacteriaFTTs]}];
bacteriaFTTsFlat = Flatten[bacteriaFTTs, 1];
generaFlat = Flatten[generaLists];
```

Create the kd tree and process the lookup vectors.

```
keep = 40;
AbsoluteTiming[{nf, vv} = imageKNN[bacteriaFTTsFlat, generaFlat, keep];]
AbsoluteTiming[testVecs = processInput[phageFTTs, vv];]
```

Get the 29 nearest bacteria chunks for each viral genome. Take all bacteria genera as guesses that appear at least twice in each such list. This gives an average of 3.0 guesses per viral genome.

```
nbrCount = 29;
Timing[nearSegments = Map[nf[#, nbrCount] &, testVecs];]
hostTops = Map[ReverseSortBy[#, Function[{x}, x[[2]]]] &, Map[Tally, nearSegments]];
Count[hostTops, {{_, a_}, ___} /; a ≥ 3]
Count[hostTops, {{_, a_}, ___} /; a ≥ 2]
hostTopsb = Map[Select[#[[2]] ≥ 2 &], hostTops];
Union[Map[Length, hostTopsb]]
Tally[Map[Length, hostTopsb]]
Mean[N[Map[Length, hostTopsb]]]
```

Check results for all guesses that appear at least twice in a given viral genome.

```
hostTopsb = Map[Select[#[[2]] ≥ 2 &], hostTops];
Union[Map[Length, hostTopsb]]
Tally[Map[Length, hostTopsb]]
Mean[N[Map[Length, hostTopsb]]]
```
